# A novel protein Moat prevents ectopic epithelial folding by limiting Bazooka/Par3-dependent adherens junctions

**DOI:** 10.1091/mbc.E24-04-0177

**Published:** 2024-07-22

**Authors:** Lingkun Gu, Rolin Sauceda, Jasneet Brar, Ferdos Fessahaye, Minsang Joo, Joan Lee, Jacqueline Nguyen, Marissa Teng, Mo Weng

**Affiliations:** a School of Life Sciences, University of Nevada, Las Vegas, NV 89154; b Department of Molecular Biology, Princeton University, Princeton, NJ 08544; University of Texas, Austin

## Abstract

Contractile myosin and cell adhesion work together to induce tissue shape changes, but how they are patterned to achieve diverse morphogenetic outcomes remains unclear. Epithelial folding occurs via apical constriction, mediated by apical contractile myosin engaged with adherens junctions, as in Drosophila ventral furrow formation. While it has been shown that a multicellular gradient of myosin contractility determines folding shape, the impact of multicellular patterning of adherens junction levels on tissue folding is unknown. We identified a novel Drosophila gene *moat* essential for differential apical constriction and folding behaviors across the ventral epithelium which contains both folding ventral furrow and nonfolding ectodermal anterior midgut (ectoAMG). We show that Moat functions to downregulate polarity-dependent adherens junctions through inhibiting cortical clustering of Bazooka/Par3 proteins. Such downregulation of polarity-dependent junctions is critical for establishing a myosin-dependent pattern of adherens junctions, which in turn mediates differential apical constriction in the ventral epithelium. In *moat* mutants, abnormally high levels of polarity-dependent junctions promote ectopic apical constriction in cells with low-level contractile myosin, resulting in expansion of infolding from ventral furrow to ectoAMG, and flattening of ventral furrow constriction gradient. Our results demonstrate that tissue-scale distribution of adhesion levels patterns apical constriction and establishes morphogenetic boundaries.

## Abstract (executive summary)

How cellular machinery activities are patterned to achieve diverse tissue shapes remains poorly understood. This study focuses on the role of tissue-wide patterning of the level of cell adhesion on epithelial folding.

The authors identified a novel gene *moat* essential for the differential apical constriction and folding behavior across tissues. Moat functions to downregulate the polarity-dependent adherens junctions through inhibiting cortical Bazooka cluster formation.

The study demonstrates that not only the presence but the level of adherens junctions is critical for apical constriction, and establishing specific patterns of junction levels promotes proper shape and boundary of epithelial folding.

## INTRODUCTION

During morphogenesis, cell shape changes exhibit precise tissue boundaries due to tissue-specific activities of ubiquitously expressed cellular machineries, most prominently contractile myosin and cell adhesion structures (Martin and Goldstein, 2014). While contractile actomyosin generates physical force, cell adhesion often provides anchors for transmitting this force to cell cortex and maintains tissue integrity. It has been shown that a multicellular gradient of contractile myosin is essential for the differential cell shape changes across the tissue (Heer *et al.*, 2017), how tissue-scale patterning of cell adhesion levels impacts the morphogenetic landscape is unknown.

A fundamental type of morphogenetic movement is epithelial folding which bends flat epithelial sheets to form complex structures. It is essential for the folding to only involve cells of correct fates, since the process often serves to internalize specific tissues such as *Caenorhabditis elegans* endoderm precursors and *Drosophila* ventral furrow, or to form tubular structures, such as Drosophila salivary gland and vertebrate neural tube. In the case of *Drosophila* ventral furrow, the region undergoing folding is patterned by the expression of transcription factors Snail (Sna) and Twist (Twi) (Leptin and Grunewald, 1990). Sna is a conserved transcription factor promoting epithelial–mesenchymal transition in mesoderm at a later stage (Thiery *et al.*, 2009) but during ventral furrow formation Sna functions to activate contractile myosin (Manning *et al.*, 2013).

**FIGURE 1:**
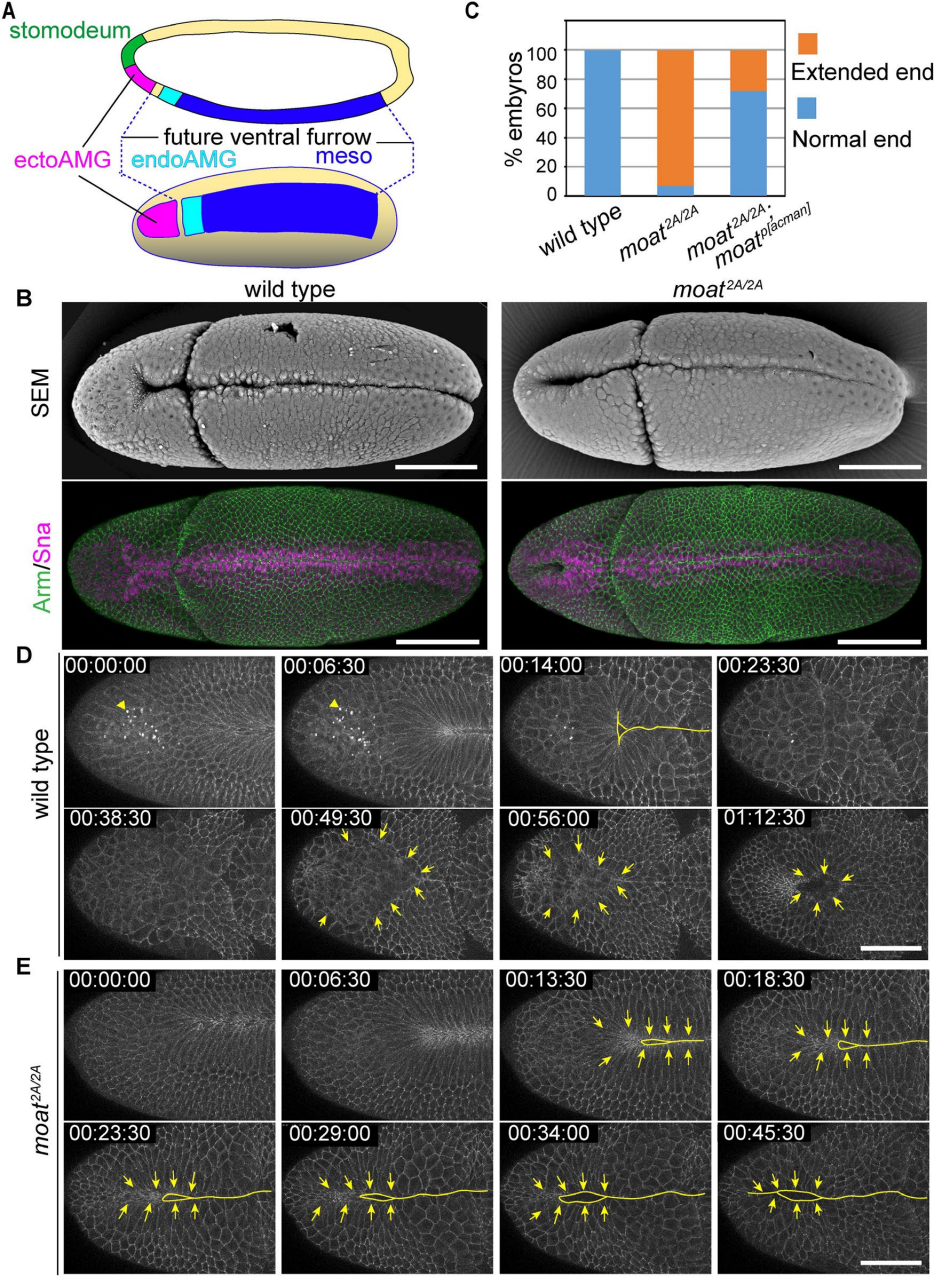
*moat* is required to prevent ectopic epithelial folding in ectoAMG. (A) Diagram illustrating different segments within Sna-expressing zone as well as the stomodeum in blastoderm. Top: Lateral view; Bottom: Ventral view. (B) SEM (top) and immunostained (bottom) images of wild-type and *moat^2A/2A^* embryos at stage 6. Scale bar: 100 µm. (C) Quantification of extended furrow phenotype. Wild type: *N* = 100; *moat^2A/2A^*: *N* = 66; *moat^2A/2A^*, Moat^p[acman]^: *N* = 90. (D) Still images from a time-lapse movie show the internalization of wild-type ectoAMG. Arrowheads in top panels: autofluorescence of yolk vesicles. Solid lines: ventral furrow. Arrow: ectoAMG boundaries. (E) Still images from a time-lapse movie show the ectopic infolding of *moat^2A/2A^* ectoAMG. Solid lines: epithelial folds. Arrows: directions of cell movements. Scale bar in D and E: 50 µm. T = hh:mm:ss

However, not all cells in Sna-expressing ventral cells participate in ventral furrow formation. This zone is thought to cover at least three tissues from anterior to posterior (Figure 1A): the ectoderm-origin anterior midgut (we refer to it as “ectoAMG”), the endoderm-origin anterior midgut (we refer to it as “endoAMG”), and the mesoderm (Hartenstein *et al.*, 1985; Technau and Campos-Ortega, 1985; Reuter and Leptin, 1994a). While endoAMG and mesoderm form the ventral furrow, ectoAMG is not involved. The lack of infolding in ectoAMG is due to the expression of patterning gene *huckebein* (*hkb*), a terminal gap gene that inhibits certain functions of Sna at embryo anterior (Reuter and Leptin, 1994a). However, it remains unknown how the activities of contractile myosin and cell adhesion differ between these tissues and how this leads to morphogenetic differences.

The ventral furrow forms through contractile myosin-mediated apical constriction. The ubiquitously expressed nonmuscle myosin II is specifically activated on apical cortices of ventral furrow cells through two parallel pathways. Components of these pathways, such as *mist*, *fog*, and *T48,* are expressed under the control of Sna and Twi (Costa *et al.*, 1994; Kölsch *et al.*, 2007; Manning *et al.*, 2013). In contrast to tissues where myosin forms junctional cables to define tissue boundaries (Röper, 2012; Chung *et al.*, 2017; Yu *et al.*, 2021), ventral furrow cells mainly organize contractile myosin into a network on medial apical cortex which constricts apical area (Martin *et al.*, 2009). This apical constriction induces cell and tissue shape changes, leading to the infolding of ventral furrow epithelium. The activity of myosin is patterned as a gradient along the ventral-lateral axis: high in cells around the ventral midline and low in the more flanking mesoderm (Heer *et al.*, 2017). This gradient is important for a coordinated ventral furrow formation. The lack of infolding in ectoAMG suggests myosin activation may also be patterned along the anterior-posterior axis of the Sna-expressing zone.

The second crucial component for apical constriction is adherens junctions, the E-Cadherin (E-Cad)-catenin–based cell-cell junctions that physically connect neighbor cells. Adherens junctions play multiple essential roles for morphogenetic events. One key function is to serve as anchors for contractile myosin, transmitting force to cell cortex (Sawyer *et al.*, 2009; Martin *et al.*, 2010; Roh-Johnson *et al.*, 2012). During fly ventral furrow formation, adherens junctions connect actomyosin filaments into a tissue-wide network. Additionally, adherens junctions promote cell shape changes without myosin when differentially positioned (Wang *et al.*, 2012). Lastly, adherens junctions are often required to maintain tissue integrity and prevent tissue rupture caused by myosin-generated tension (Martin *et al.*, 2010; Razzell *et al.*, 2018).

While the presence of adherens junctions is essential for various morphogenetic events, it remains unknown whether the level of adherens junctions plays a role in modulating the boundaries of cell shape changes. Cadherin–Catenin complexes interact laterally to form clusters, which are the basic unit of adherens junctions (Strale *et al.*, 2015; Wu *et al.*, 2015). Here, we define the level of adherens junctions by combining the number, size, and density of the lateral clusters of Cadherin–Catenin complexes. In early fly embryos, adherens junction clusters form many micron-sized puncta called spot adherens junctions (Cavey *et al.*, 2008; Tepass and Hartenstein, 1994). The number, size, and E-Cad density of these puncta change as the embryo develops. The levels of these spot adherens junctions can be patterned subcellularly to direct morphogenesis. For example, the planar polarized adherens junctions have been shown to orient the directional myosin flow and drive convergent extension (Rauzi *et al.*, 2010).

The level of adherens junctions can also be patterned across the tissue. Although fly ventral furrow cells require strong junctions for successful apical constriction, they are specifically patterned to start apical constriction with low levels of junctions (Weng and Wieschaus, 2017). The benefit of this patterned low level of junctions is poorly understood, but the molecular mechanism involves a Sna-dependent downregulation of junctional Bazooka (Baz), the fly polarity protein Par-3. Par-3 plays a critical role in the assembly, stability, and positioning of adherens junctions across various systems (Harris and Peifer, 2004; Harris and Peifer, 2005; Achilleos *et al.*, 2010; Simões *et al.*, 2010). During the initial polarization of the single-layer epithelium in early fly embryo, Baz localizes to future junctional sites, which is essential for the assembly of adherens junctions (Harris and Peifer, 2004; Harris and Peifer, 2005). However, shortly before gastrulation, junctional Baz starts to be downregulated in mesoderm, leading to low levels of adherens junctions immediately prior to apical constriction (Weng and Wieschaus, 2016; Weng and Wieschaus, 2017). Sna is necessary and sufficient for this downregulation, but it is not clear whether the downregulation occurs in all Sna-expressing cells.

Although these mesoderm cells do regain strong junctions during apical constriction via a myosin-dependent mechanism (Weng and Wieschaus, 2016), an important question remains: does this tissue-specific reduction in junction levels immediately prior to apical constriction contribute to the spatial pattern of apical constriction and the boundaries of the ventral furrow formation? To understand the significance of Baz and junction downregulation, we characterized a novel Drosophila protein, loss of which results in abnormally high levels of junctional Baz and adherens junctions. This disrupts the low level of Baz-dependent junctions patterned in Sna-expressing zone and leads to extension of ventral furrow infolding behavior to the neighbor tissue.

## RESULTS

### *moat* mutant embryos show ectopic epithelial folding of ectoAMG

To understand tissue boundary formation, we identified an uncharacterized gene *CG14427* through a compound-chromosome genetic screen looking for mutants with disrupted organization of ventral furrow. In wild-type embryos, the most anterior end of Sna-expressing zone, ectodermal anterior midgut (ectoAMG), does not participate in the ventral furrow. The rest of the Sna-expressing zone, including mesoderm and endodermal anterior midgut (endoAMG), is folded in as the ventral furrow. The anterior end of ventral furrow forms a Y-shaped structure at about 15 percentile egg length. (Figure 1, A and B, wild type [Hartenstein *et al.*, 1985; Technau and Campos-Ortega, 1985; Reuter and Leptin, 1994a]). Strikingly, in the *CG14427* mutant embryos, we observed that the infolding behavior of ventral furrow appears to be extended to the very anterior end of the embryo and include the presumptive ectoAMG (Figure 1, B and C). We named this gene *moat* for its role in safeguarding the tissue morphogenetic boundaries, and generated a CRISPR allele (*moat^2A^*) deleting majority of the coding region (Supplemental Figure S1A). A small genomic fragment containing this gene restores the normal position of ventral furrow end (Figure 1C). Consistent with ectoAMG showing the most obvious defects in *moat* mutant embryo, we found that *moat* mRNA is most prominently detected in ectoAMG while its expression in other tissues is comparably more transient (Supplemental Figure S1B). Moat protein is expressed in a similar pattern as its mRNA (Supplemental Figure S1C). Moat protein consists of 252 amino acids and its homologues are found in the Brachyceran suborder of Dipteran insects. Although there is not any recognizable functional domain, Moat protein appears to associate with cell membrane or cortex (Supplemental Figure S1, C and D).

To understand the ectoAMG phenotype in *moat* mutants, we first characterized the invagination of ectoAMG in wild-type embryos which has not been studied. We used E-Cad::GFP which labels spot adherens junctions as puncta and cell membrane as dim lines. In wild-type embryos, ectoAMG cells are excluded from the Y-shaped end of ventral furrow and remain on the surface of the embryo (Figure 1D; Supplemental Movie S1). About 25–30 min after the ventral furrow closure, ectoAMG starts its own course of internalization through a different mechanism. The ectoAMG boundary can be easily distinguished due to the brighter adherens junctions in neighboring ectoderm (Figure 1D, arrows). The boundary remains mostly circular, but the encircled surface area gradually decreases. ectoAMG cells are completely internalized in about 40 min, leaving a temporary circular opening on the embryo surface (Figure 1D, last panel). This is in contrast to the past description that ectoAMG participates in the triangle-shaped stomodeum invagination (Hartenstein *et al.*, 1985; Technau and Campos-Ortega, 1985; Reuter and Leptin, 1994a). Our live imaging data show that the triangular stomodeum invagination is a separate event: it consists of cells anterior to ectoAMG and is initiated later than the circular ectoAMG invagination (Supplemental Movie S2). The triangle-shaped invagination also features much higher levels of adherens junctions compared with ectoAMG, making the two tissues easily distinguishable. Altogether, our data indicate that the internalization of wild-type ectoAMG is a morphogenetic event different from that of ventral furrow and stomodeum in both morphology and timing.

**Figure:** Movie S1

**Figure:** Movie S2

In contrast, the presumptive ectoAMG in *moat* mutants does not stay on embryo surface during ventral furrow formation but is internalized as a linear fold continuous with ventral furrow (Figure 1E; Supplemental Movie S3). Not only is this internalization earlier than that in wild-type embryos, but it is also morphologically different. These mutant cells are pulled medially, perpendicular to the ventral midline, a movement similar to ventral furrow cells (Figure 1E, arrows). This is distinct from the circular invagination of the wild-type ectoAMG. In summary, *moat* mutant presumptive ectoAMG undergoes an ectopic infolding continuous with ventral furrow and as a result is internalized prematurely.

**Figure:** Movie S3

### The ectopic folding of ectoAMG in *moat* mutants is not due to aberrant expression of early patterning genes

Next, we investigated whether the ectopic infolding in *moat* mutants is due to defects in early patterning gene expression. We chose to examine two gap genes *giant* (*gt*) and *hkb* because previous studies suggest their expression patterns likely define the boundary between ectoAMG and ventral furrow cells (Eldon and Pirrotta, 1991; Reuter and Leptin, 1994b). Because the spatial relationship between the expression of these proteins and the morphogenetic movement has not been studied at cellular levels in AMG region, we first characterized the expression of Gt and Hkb in ectoAMG and endoAMG in wild-type embryos.

**FIGURE 2:**
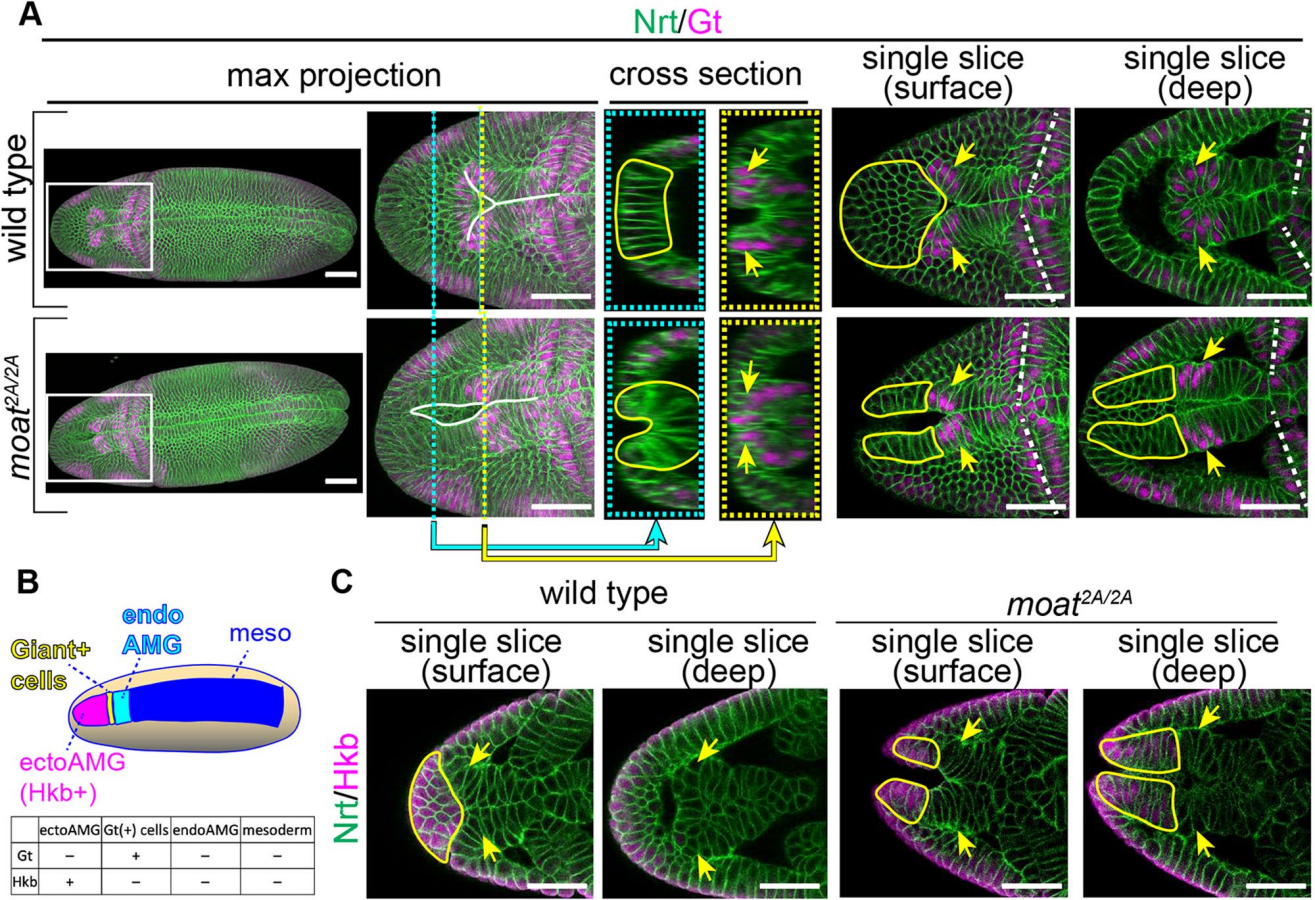
Patterning genes Gt and Hkb are expressed normally in *moat* mutant embryos. (A) Wild-type and *moat^2A/2A^* embryos immunostained for Gt and Nrt (membrane). White lines in max projections: epithelial folds. Cross-section images are transverse views (YZ) generated from confocal stacks (XYZ) using the Reslice function in Fiji at the X positions indicated by the dashed cyan and yellow lines. Single slice images are single optical sections from confocal stacks. Enclosed yellow lines: presumptive ectoAMG based on morphology and position. Yellow arrows: Gt(+) cells. White dashed lines: cephalic furrows. Scale bar: 50 µm. (B) Diagram showing four distinct tissues in Snail expression zone. (C) Single optical sections of anterior regions of embryos immunostained for Hkb and Nrt. Enclosed yellow lines: Hkb(+) ectoAMG. Arrows: presumptive Gt(−) cells. Scale bar: 50 µm.

In wild-type embryos, Gt is expressed in a narrow stripe on the ventral side of the head region, in addition to the well-studied lateral stripes (Eldon and Pirrotta, 1991). We further found that this ventral anterior Gt stripe is situated as a narrow segment in the Sna-expressing zone at blastoderm stage (Supplemental Figure S2A). To determine whether Gt is expressed in ectoAMG or endoAMG, we focused on the stage when the Y-shaped end of ventral furrow becomes apparent so we can use morphologic features to identify different tissues. Our live imaging data (Figure 1D) show that, at this stage, wild-type ectoAMG resides on embryo surface immediately anterior to the Y-shaped structure. In immunostained embryos of the same stage, we observed that the ventral anterior Gt stripe appears to surround the Y-shaped structure in max projection images, giving the impression that ectoAMG cells immediately anterior to the Y shape would be Gt(+) (Figure 2A, wild type, max projection). However, single optical sections reveal that the Gt(+) signals overlapping with ectoAMG in max projections are not from ectoAMG. They are from the internalized ventral furrow cells detected only in deeper optical slices (Figure 2A, wild type, yellow arrows). ectoAMG cells, which remain on embryo surface, are all Gt(−) (Figure 2A, wild type, encircled by yellow lines)

These data indicate that Gt(+) cells are not ectoAMG but constitute the anterior end of the internalized ventral furrow. The anterior end of the ventral furrow was thought to be endoAMG based on horseradish peroxidase labeling experiments (Hartenstein *et al.*, 1985; Reuter and Leptin, 1994a), which would suggest the Gt(+) cells are endoAMG. However, by following Gt(+) cells in later stage embryos, we found that they do not contribute to AMG structure: they remain in the head region, and spread to the lateral side (Supplemental Figure S2B). This is consistent with the previous study on Gt expression (Eldon and Pirrotta, 1991). Taken together, we concluded that the anterior ventral Gt(+) cells are an uncharacterized tissue positioned as a segment in Sna-expressing zone at blastoderm stage between ectoAMG and endoAMG primordia (Figure 2B).

**FIGURE 3:**
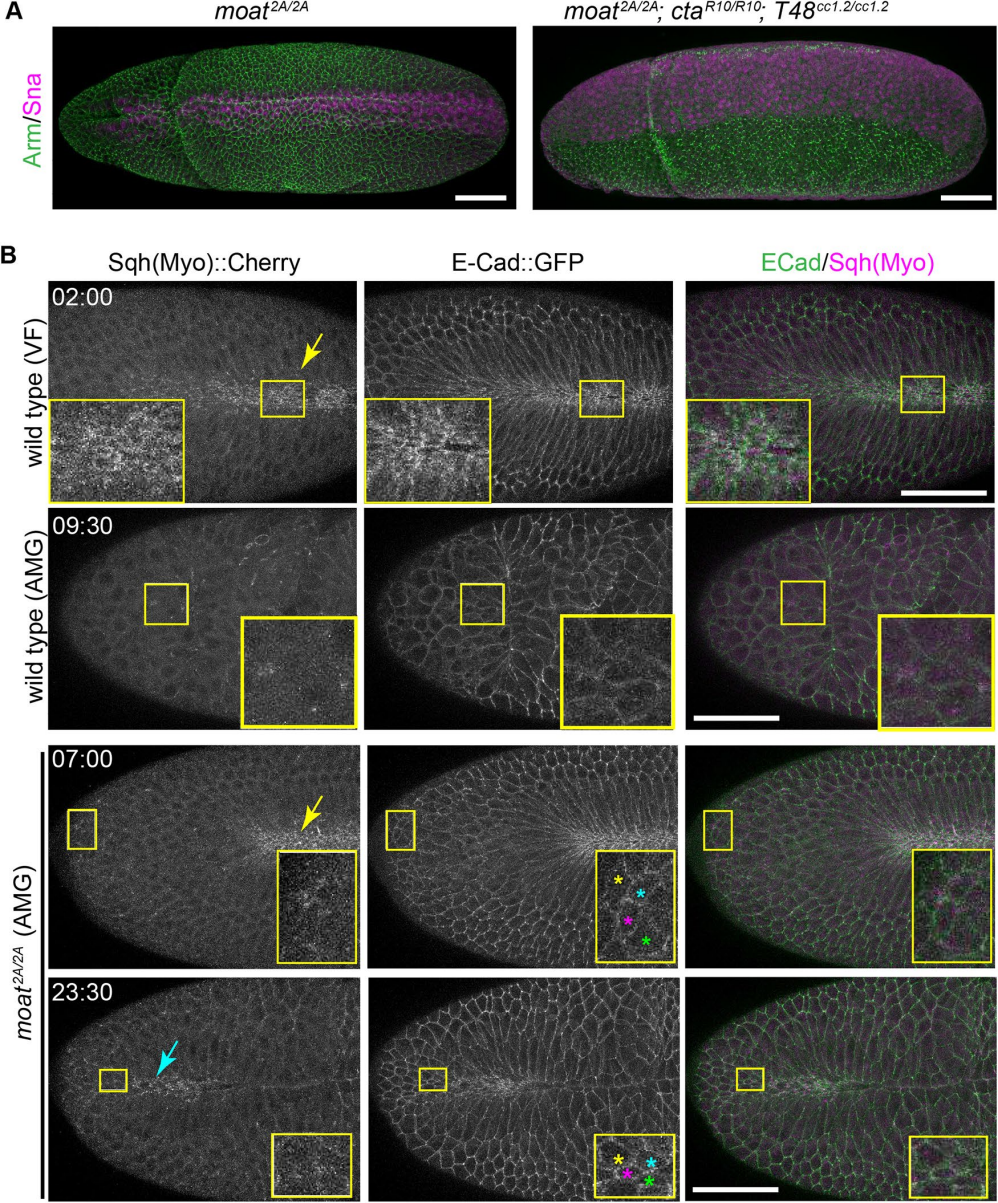
Ectopic apical constriction in *moat* mutant ectoAMG. (A) Immunostained embryos of stage 7. (B) Still images from time-lapse movies of indicated genotypes and tissue types. Inserts: enlarged images of the boxed regions. Colored stars: tracked cells at the two timepoints. Yellow arrows: active myosin in wild-type or *moat^2A/2A^* ventral furrows. Cyan arrow: active myosin in highly constricted cells in *moat^2A/2A^* ectoAMG. Scale bar in A and B: 50 µm. T = mm:ss

Next, we examined the expression of another gap gene *hkb* in wild-type embryos. We found that Hkb protein is expressed in the presumptive ectoAMG cells but not the Gt(+) cells (Figure 3C). Single optical slices show that Hkb is detected in ectoAMG cells occupying the surface position immediately anterior to the Y-shape structure (Figure 2C, wild type, encircled by the yellow line). However, Hkb is absent in the presumptive Gt(+) cells identified as the anterior end of internalized ventral furrow (Figure 2C, wild type, yellow arrows). Taken together, our data showed that there are four different segments from anterior to posterior in wild-type Sna-expressing zone: 1) Gt(−) Hkb(+) ectoAMG; 2) the uncharacterized Gt(+) Hkb(−) cells; 3) Gt(−) Hkb(−) endoAMG; 4) Gt(−) Hkb(−) mesoderm (Figure 2B).

In *moat* mutant embryos, neither Gt nor Hkb expression pattern appears to be altered. The ventral Gt stripe remains at about 15 percentile egg length similar to that in wild-type embryos. The elongated mutant furrow passes through the Gt stripe to include the anterior Gt(−) cells (Figure 2A, *moat^2A/2A^*, max projection). These Gt(−) cells often form an incomplete tube with a narrow opening on the embryo surface which is continuous with the epithelial tube formed by ventral furrow (Figure 2A, *moat^2A/2A^*, cyan cross-section). As a result, Gt(+) cells appear as a segment of the continuous tube (Figure 2A, *moat^2A/2A^*, deep single slice, cross-sections). Similarly, *moat* mutant embryos express Hkb normally. Hkb remains to be expressed in the anterior ventral cells despite their ectopic infold in *moat* mutants (Figure 2C, *moat^2A/2A^*, encircled by yellow lines). Hkb is also not detected in the presumptive Gt(+) cells in *moat* mutant embryos identified by their positions, same as the wild type (Figure 2C, *moat^2A/2A^*, yellow arrows).

These data indicate that the ectopic infolding observed in *moat* mutant embryos is not due to an expansion of ventral furrow fate to the anterior end of the embryo, and ectoAMG are most likely specified properly in *moat* mutant embryos.

### ectoAMG in *moat* mutant embryos exhibit ventral furrow-like apical constriction

Because ventral furrow formation is driven by myosin-mediated apical constriction, we investigated whether the ectopic infolding of ectoAMG in *moat* mutant embryos is driven by the same mechanism. During ventral furrow formation, myosin is activated by two parallel pathways that recruits RhoGEF2 to apical cortex: one mediated by a transmembrane G protein Cta (Costa *et al.*, 1994; Barrett *et al.*, 1997; Dawes-Hoang, 2005; Manning *et al.*, 2013) and the other by an apical membrane-localized protein T48 (Kölsch *et al.*, 2007). Eliminating both *cta* and *T48* completely abolishes apical constriction in ventral furrow.

First, we tested whether the loss of *cta* and *T48* would suppress the ectopic apical constriction of *moat* mutant ectoAMG. Indeed, in *moat, cta*, *and T48* triple mutant embryos, not only ventral furrow but also ectoAMG fail to undergo infolding and are left on the embryo surface (Figure 3A). This suggests that the ectopic infolding in *moat* mutant ectoAMG requires the same pathways that induce contractile myosin in wild-type ventral furrow.

Next, we examined whether contractile myosin can be detected in ectoAMG. Past studies indicate that contractile myosin can be detected as the intense and pulsatile cortical myosin structures that are distinguishable from the uniformly distributed inactive myosin in the cytoplasm, and its intensity correlates with apical constriction (Xie and Martin, 2015; Vasquez *et al.*, 2016; Heer *et al.*, 2017). We refer to this population of myosin as contractile myosin and compared three conditions: 1) wild-type ventral furrow region, known to undergo robust myosin-mediated infolding; 2) wild-type ectoAMG, a tissue that does not undergo infolding; 3) *moat* mutant ectoAMG that undergoes ectopic infolding. Wild-type ventral furrow cells display strong contractile myosin on the apical cortex, and undergo apical constriction and infolding (Figure 3B, top row; Supplemental Movie S4). By contrast, wild-type ectoAMG cells display detectable but low levels of pulsatile myosin and exhibit little apical constriction (Figure 3B, second row; Supplemental Movie S5). Compared with wild-type ectoAMG, *moat* mutant ectoAMG cells appear to exhibit slightly more intense and pulsatile myosin, which constrict their apical surfaces: the apical areas of the tracked cells reduce as they form the ectopic fold (Figure 3B, bottom two rows). The increase in myosin activities in mutant ectoAMG compared with wild type is more noticeable in highly constricted cells (Figure 3B, bottom row, cyan arrow; Supplemental Movie S6) but nevertheless much weaker than that in the ventral furrows of either wild type or *moat* mutant embryos (Figure 3B, yellow arrows).

**Figure:** Movie S4

**Figure:** Movie S5

**Figure:** Movie S6

### The increase in myosin activities in *moat* mutant ectoAMG is mild

**FIGURE 4:**
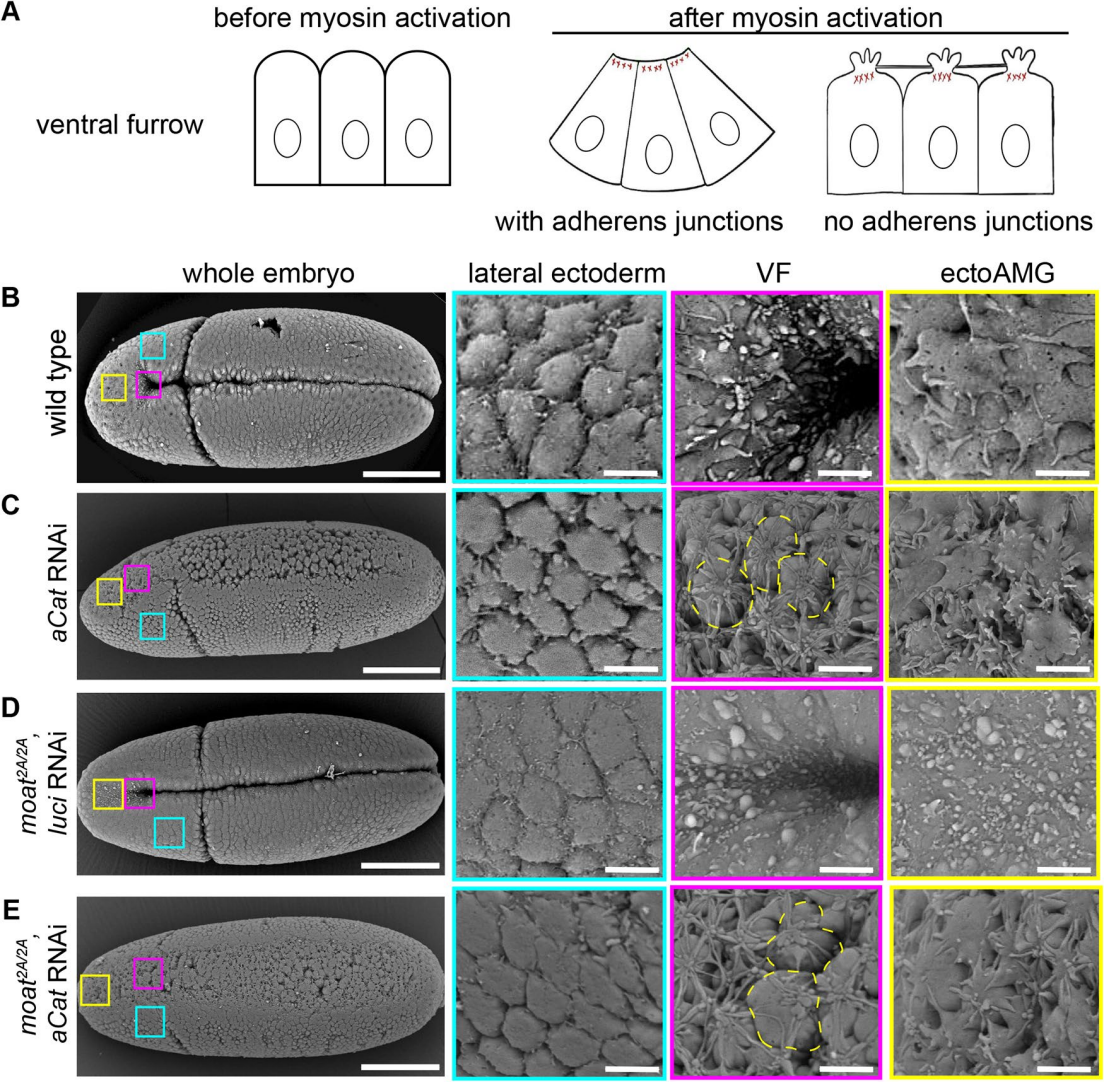
*moat* mutant ectoAMG does not resemble ventral furrow in the complete cell rupture phenotype upon loss of junctions. (A) Diagram illustrating the morphological changes of the apical surface in response to contractile myosin. (B–E) SEM images of embryos of indicated genotypes. High magnification images of specific tissues boxed in the whole embryo are displayed on the right. Yellow dashed circles: individual cells ruptured from neighbors, displaying the membrane foci and tethers. Scale bar in B–E: 100 µm for whole embryos images and 5 µm for high magnification images.

The above comparison of myosin could be confounded by apical constriction occurring only in *moat* mutant but not wild-type ectoAMG cells, since contractile myosin can appear more concentrated in cells with reduced apical area. To compare myosin activities without this complication, we disrupted apical constriction by depleting myosin’s anchors to the cell cortex: adherens junctions. Without adherens junctions, apical constriction cannot occur, but myosin activity can be assessed by observing its impact on apical membrane deformation through scanning electron microscopy (SEM) (Martin *et al.*, 2010) (Figure 4A).

Apical membrane deformations as a response to different levels of contractile myosin have been well characterized in ventral furrow cells in both wild-type and adherens junction-defective embryos (Sweeton *et al.*, 1991; Martin *et al.*, 2010) (Supplemental Figure S3). Before myosin activation, all cells display smooth and dome-shaped apices with well-defined cell boundaries (Supplemental Figure S3A). As embryos develop to gastrulation, lateral ectoderm retains the dome shaped apices since myosin is not activated in this tissue (Figure 4B, lateral ectoderm; Supplemental Figure S3, A–C). However, in ventral furrow cells, apical membrane morphology shows distinct changes in response to changing levels of myosin contraction. Initially, low levels of myosin contraction lead to flattened apical surfaces resulting in less distinct cell boundaries (Supplemental Figure S3B). Later, strong myosin contraction leads to invisible cell boundaries, and accumulation of small membrane blebs (Figure 4, A and B; Supplemental Figure S3C).

In junction-defective embryos such as α-Catenin (α-Cat) RNAi embryos, the nonconstricting ectoderm cells still maintain dome-shaped apices (Figure 4C, lateral ectoderm). However, ventral furrow cells in the same embryo display a dramatic deviation from their wild-type counterparts: complete ruptures occur between cell apices due to high levels of myosin contraction without adherens junctions (Figure 4C, VF). As a result, the apical cortices in individual cells contract independently, leading to apical membrane accumulation in one focus on top of each cell. These membrane foci are connected by long membrane tethers due to the residual cell adhesion between cells (Figure 4, A and C, VF).

With these known responses of apical membrane to contractile myosin, we examined the apical membrane morphology in ectoAMG of different genotypes. First, we examined ectoAMG in wild-type background with or without α-Cat RNAi. Although wild-type ectoAMG cells do not undergo apical constriction, their apices appear to be flattened (Figure 4B, ectoAMG), resembling the response of ventral furrow cells to low-level myosin contraction (Supplemental Figure S3B). This is consistent with the low-level contractile myosin in wild-type ectoAMG (Figure 3B). In α-Cat RNAi embryos, ectoAMG displays small cell tears and some membrane tethers, but the degree of deformation is much less than that in the ventral furrow: no membrane foci can be observed (Figure 4C, ectoAMG vs. VF). This suggests wild-type ectoAMG cells have low levels of contractile myosin that leads to small cell tears when adherens junctions are depleted.

Next, we examined *moat* mutant embryos with or without α-Cat RNAi. In *moat* embryos with control luciferase RNAi (*Luci* RNAi), lateral ectoderm and ventral furrow do not show significant changes in apical membrane morphology compared with wild type: lateral ectoderm still shows smooth apices with well-defined cell boundaries and ventral furrow cells still show small membrane blebs with invisible cell boundaries (Figure 4D vs. 4B, lateral ectoderm, VF). Similarly, these two tissues with α-Cat RNAi in *moat* mutant background resemble their counterpart tissues with α-Cat RNAi in wild-type background: ectoderm maintains smooth apices and clear cell boundaries while ventral furrow displays complete cell ruptures with membrane foci and long membrane tethers (Figure 4E vs. 4C, lateral ectoderm, VF).

By contrast, *moat* mutant ectoAMG differs significantly from its wild-type counterpart in apical membrane morphology. *moat* mutant ectoAMG displays apical membrane features similar to that of ventral furrow with strong contractile myosin: unrecognizable boundaries and small membrane blebs on apical surfaces (Figure 4D, ectoAMG; Supplemental Figure S3C). This is consistent with *moat* mutant ectoAMG exhibiting ventral furrow-like apical constriction behavior. However, *moat* mutant ectoAMG responds differently from ventral furrow when α-Cat is further removed. ectoAMG with α-Cat RNAi in *moat* mutant background lacks the complete cell rupture phenomenon observed in ventral furrow with α-Cat RNAi: there are no membrane foci on top of individual cells but only small cell tears and tethers (Figure 4E ectoAMG vs. Figure 4C VF or 4E VF). This phenotype is more similar to ectoAMG with α-Cat RNAi in wild-type background, except one detail: the small membrane tethers in *moat* mutant background appear to be straighter and reaching to neighboring cells more, suggesting moderately increased contractile myosin in *moat* mutant ectoAMG (Figure 4E vs. 4C, ectoAMG).

Taken together, we conclude that, despite the similar apical constriction behavior, the myosin activities in *moat* mutant ectoAMG is much lower than that in ventral furrow and is only moderately increased compared with wild-type ectoAMG. We also showed that adherens junctions are required for *moat* mutant ectoAMG to exhibit the ventral furrow-like morphology such as reduction in apical area and apical membrane blebs.

### *moat* mutant embryos display abnormally high levels of Baz/Par3-dependent adherens junctions

How does the low-level contractile myosin in *moat* mutant ectoAMG achieve morphogenetic outcomes similar to the strong contractile myosin in ventral furrow? The other component of apical constriction is adherens junctions which engage contractile myosin for force transmission. Therefore, we hypothesized that *moat* mutants may display abnormal adherens junctions that promote ectopic folding with low levels of contractile myosin.

**FIGURE 5:**
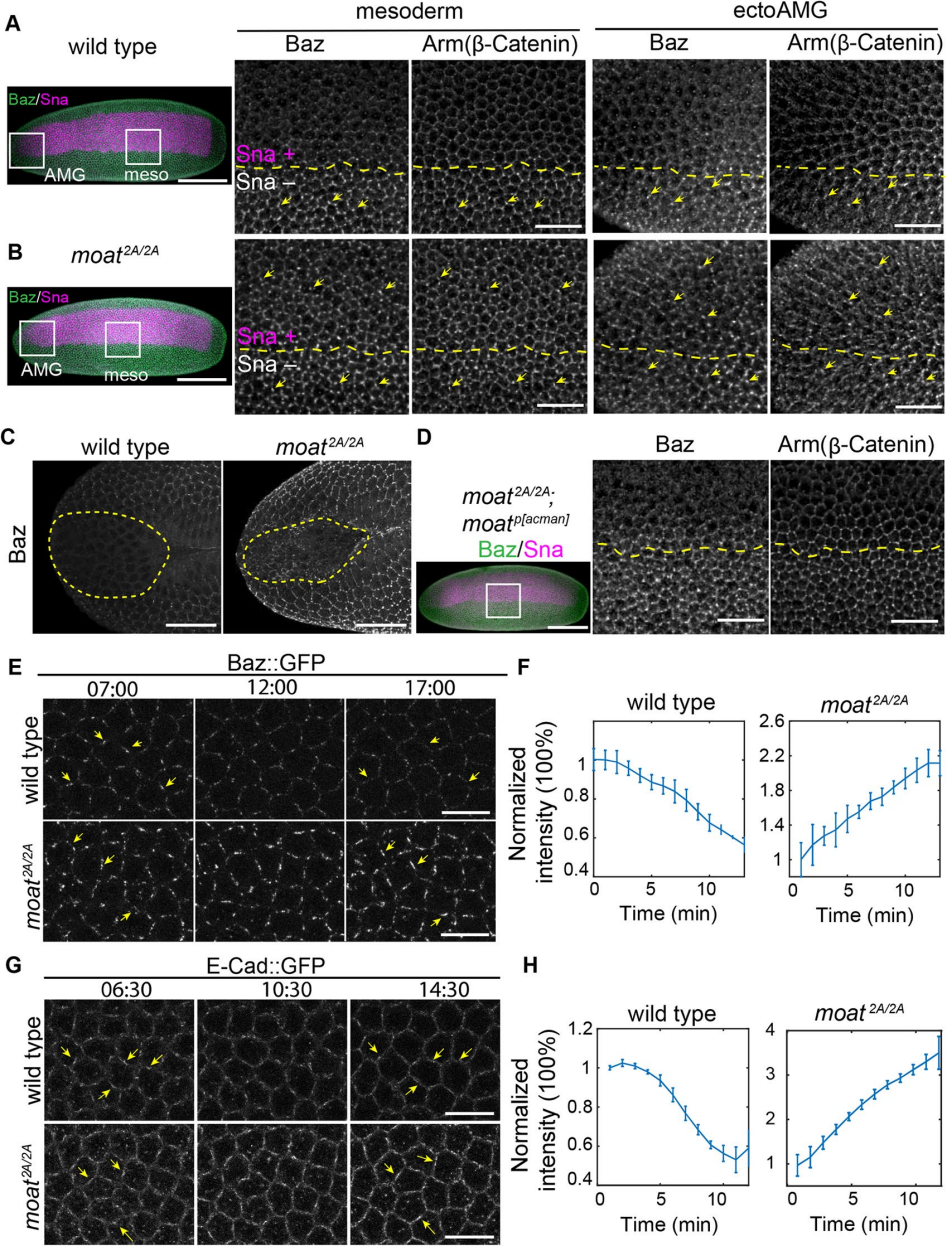
Moat disrupts Sna-dependent downregulation of junctional Baz and adherens junctions. (A, B) Late stage 5, wild type (upper panels) and *moat^2A/2A^* (lower panels) embryos were immunostained for Baz, Arm and Sna. Left: whole embryo’s max projection images; Right: enlarged images of boxed regions in the whole embryo images, showing mesoderm/ectoAMG and their neighboring ectoderm. Yellow dashed lines: the boundaries between Sna-positive and -negative cells. Yellow arrows: Baz puncta and their corresponding junction puncta. Scale bar: 100 µm for whole embryos images and 25 µm for enlarged images. (C) Late stage 6 wild type and *moat^2A/2A^* embryos showing junctional Baz in ectoAMG. Dashed lines: ectoAMG. Scale bar: 25 µm. (D) Genomic fragment-rescued *moat^2A/2A^* embryo. Right panel: enlarged image of the boxed region. Scale bar: 25 µm. (E) Still images from time-lapse movies of Baz::GFP in mesoderm. Arrows: the same cell edges in early (left panel) and late (right panel) timepoints. Scale bar: 10 µm. (F) Quantification of Baz::GFP cluster intensity from the time-lapse movies. *N* = 3 embryos; ∼9 × 15 cells per embryo. Mean and SD (S.D.) are plotted. (G) Still images from time-lapse movies of E-Cad::GFP in mesoderm. Arrows: the same cell edges in early (left panel) and late (right panel) timepoints. Scale bar: 10 µm. (H) Quantification of E-Cad cluster intensity from the time-lapse movies. *N* = 3 embryos; ∼9 × 15 cells per embryo. Mean and S.D. are plotted. In F and H, only signals from Baz or junction puncta were quantified (Supplemental Figure S4A). For E and G: T = mm:ss

In wild-type embryos, adherens junctions are initially established as discrete puncta throughout the epithelium of fly early embryo. This depends on polarity protein Bazooka (Baz, fly Par3) clustering at subapical cortex (Harris and Peifer, 2004). Both junctional Baz and spot adherens junctions appear as micron-size puncta in early embryos and their intensity and size increase as embryos develop. However, shortly before apical constriction, junctional Baz puncta in ventral furrow cells start to decrease in response to the rising Sna level, resulting in downregulation of spot adherens junctions (Weng and Wieschaus, 2016). This downregulation creates a pronounced contrast in the number, size, and intensity of Baz and junction puncta between mesoderm and lateral ectoderm at late blastoderm stage (Figure 5A, mesoderm) (Weng and Wieschaus, 2016). Armadillo (Arm, fly β-Catenin) labels spot adherens junctions as bright puncta (Figure 5A, yellow arrow). Nonjunctional Arm labels cell membrane as dim and uniform lines. Because ectoAMG also expresses Sna, we examined whether this Sna-dependent downregulation occurs in ectoAMG. Indeed, wild-type ectoAMG exhibits a similar loss of junctional Baz and adherens junctions (Figure 5A, ectoAMG). This shows that although Sna’s function in promoting epithelial folding only occurs in ventral furrow but not in ectoAMG, its function in downregulating junctional Baz puncta occurs in all the Sna-expressing cells.

By contrast, *moat* mutant embryos fail to downregulate junctional Baz in both mesoderm and ectoAMG despite expressing Sna normally. This obscures the sharp contrast in junctional Baz levels between ectoderm and these two tissues (Figure 5B, Baz). The persistent junctional Baz leads to abnormally high levels of spot adherens junctions in both mesoderm and ectoAMG (Figure 5B, Arm). Even at a stage when ventral furrow has fully folded and wild-type ectoAMG no longer shows detectable junctional Baz, mutant ectoAMG still exhibits significant levels of junctional Baz (Figure 5C). The genomic fragment containing *moat* restores the clear contrast in junctional Baz in mesoderm (Figure 5D).

We used live imaging data to quantify the intensity change of Baz and junction puncta in wild-type and *moat* mutant mesoderm during the stage when these puncta are normally downregulated in wild-type embryos. In wild-type mesoderm, junctional Baz puncta start to diminish about 10 min before gastrulation (Figure 5, E and F; Supplemental Movie S7) (Weng and Wieschaus, 2017). In contrast, junctional Baz puncta continue to grow in size and intensity in *moat* mutant mesoderm during the same stage (Figure 5, E and F; Supplemental Movie S7). The spot adherens junctions follow a similar trend (Figure 5, G and H; Supplemental Movie S8). The loss of clear contrast in junctional Baz levels between Sna-expressing cells and the lateral ectoderm could be due to elevated levels of junctional Baz in either the Sna-expressing cells only or the entire embryo. By examining junctional Baz in ectoderm, we found that *moat* mutants exhibit higher levels of junctional Baz also in the ectoderm (Supplemental Figure S4B; Supplemental Movie S9). However, this global increase in junctional Baz is not due to higher expression of Baz protein. Western blot shows the amount of Baz protein in *moat* mutant and control embryos are not significantly different (Supplemental Figure S4C). Taken together, our data suggest that loss of *moat* enhances the tendency of Baz to form punctate structure at the junctional cortex, thereby increasing junctional localization of Baz without changing Baz protein levels.

**Figure:** Movie S7

**Figure:** Movie S8

**Figure:** Movie S9

### Junctional Baz is essential for ectopic apical constriction in *moat* mutant ectoAMG

Because *moat* mutant embryos display higher levels of junctional Baz and Baz-dependent adherens junctions, we reasoned that the high levels of Baz-dependent adherens junctions may compensate for the low levels of myosin in promoting ectopic apical constriction in *moat* mutant ectoAMG. As discussed earlier, adherens junctions in early fly embryos are formed through two mechanisms. The Baz-dependent mechanism determines the junction levels before entering apical constriction, while the myosin-dependent mechanism is essential for regaining adherens junctions in normal ventral furrow during apical constriction (Weng and Wieschaus, 2016). We hypothesized that ventral furrow formation may predominantly rely on myosin-dependent adherens junctions, while the ectopic infolding of *moat* mutant ectoAMG would require Baz-dependent adherens junctions.

**FIGURE 6:**
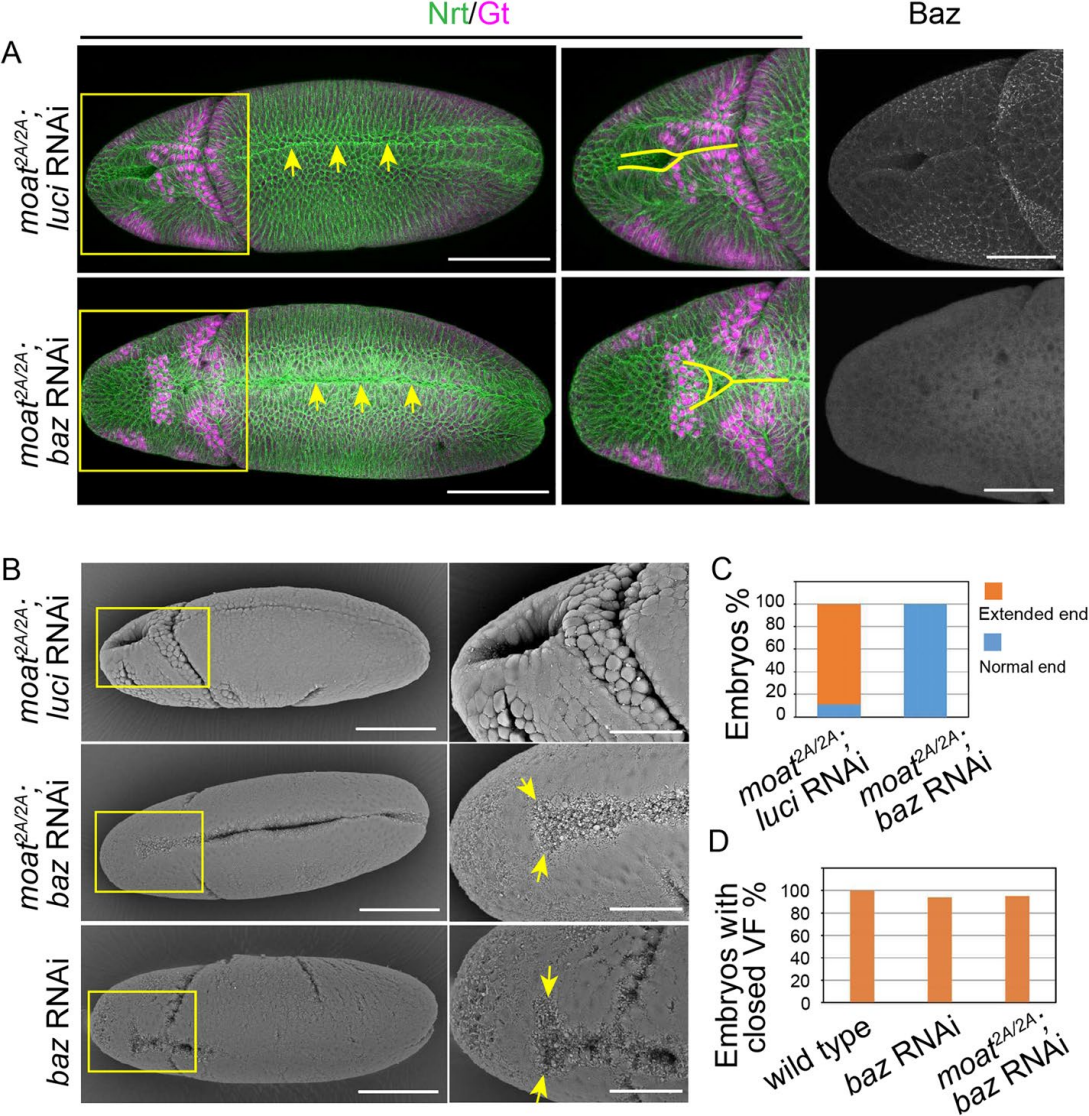
Baz is required for the ectopic epithelial folding in *moat* mutant ectoAMG. (A) Immunostained embryos with enlarged images of the boxed regions. Arrows: closed ventral furrow; Solid lines: anterior end of the fold. (B) SEM images of embryos of indicated genotypes. The enlarged images of AMG regions show the ends of the epithelial folds. Arrows: the boundary between VF and AMG region. Scale bar for A and B: 100 µm for whole embryos images and 50 µm for enlarged images. (C) Quantification of normal and extended furrow end phenotypes. *moat^2A/2A^*; *luci* RNAi: *N* = 30; *moat^2A/2A^*; *Baz* RNAi: *N* = 20. (D) Percentage of embryos with at least one-third of their middle ventral furrow closed. Wild type: *N* = 36; *Baz* RNAi: *N* = 32; *moat^2A/2A^*; *Baz* RNAi: *N* = 42.

Consistent with our hypothesis, we found that knocking down Baz abolishes the ectopic infolding of ectoAMG in *moat* mutant embryos and restores the anterior furrow end to the Gt(+) cells (Figures 2A and 6A). By contrast, Baz depletion has a much milder effect on ventral furrow. Although Baz is undetectable using immunostaining, ventral furrow still forms (Figure 6A, arrows). These phenotypes can be clearly seen using SEM. In *moat* mutant embryos with Baz RNAi, there is a clear distinction between ectoAMG and ventral furrow: ectoAMG cells are relaxed with little signs of constriction, while ventral furrow cells are strongly constricted, displaying extensive membrane blebbing (Figure 6B, arrows). The ectopic infolding of ectoAMG is lost in all *moat* mutant with Baz RNAi embryos we examined (Figure 6C). Ventral furrows, on the other hand, largely form with Baz depleted (Figure 6B, bottom two rows). The closure of the two ends of the ventral furrow tends to be incomplete, but more than 90% of embryos close at least the middle third of the ventral furrow (Figure 6D).

These results suggest that in ventral furrow, strong myosin contraction is mostly sufficient for generating both tension force and strong adherens junctions to mediate effective apical constriction and epithelial folding. Whereas, in *moat* mutant ectoAMG, although myosin contraction is slightly higher than that in wild-type ectoAMG, it is insufficient to induce ventral furrow–like apical constriction without the high levels of Baz-dependent adherens junctions. The combination of weak contractile myosin and strong Baz-dependent adherens junctions leads to the expansion of infolding behavior from ventral furrow to ectoAMG.

### Flanking mesoderm cells in *moat* mutant embryos undergo ectopic apical constriction

If the elevated Baz-dependent adherens junctions in *moat* mutants can enhance apical constriction in *moat* mutant ectoAMG, we asked whether other tissues with low-level contractile myosin would also undergo ectopic apical constriction in *moat* mutant embryos. We chose to examine the flanking mesoderm because of its low levels of contractile myosin and lack of apical constriction in wild-type embryos. Not all mesoderm cells constrict equally. Along the ventral-lateral axis of mesoderm, there is a gradient of apical constriction resulted from a gradient of contractile myosin: cells close to the midline show stronger contractile myosin and constrict their apices, while cells further from the midline show lower contractile myosin and expand their apices (Heer *et al.*, 2017). This gradient of constriction is essential for coordinated ventral furrow formation.

**FIGURE 7:**
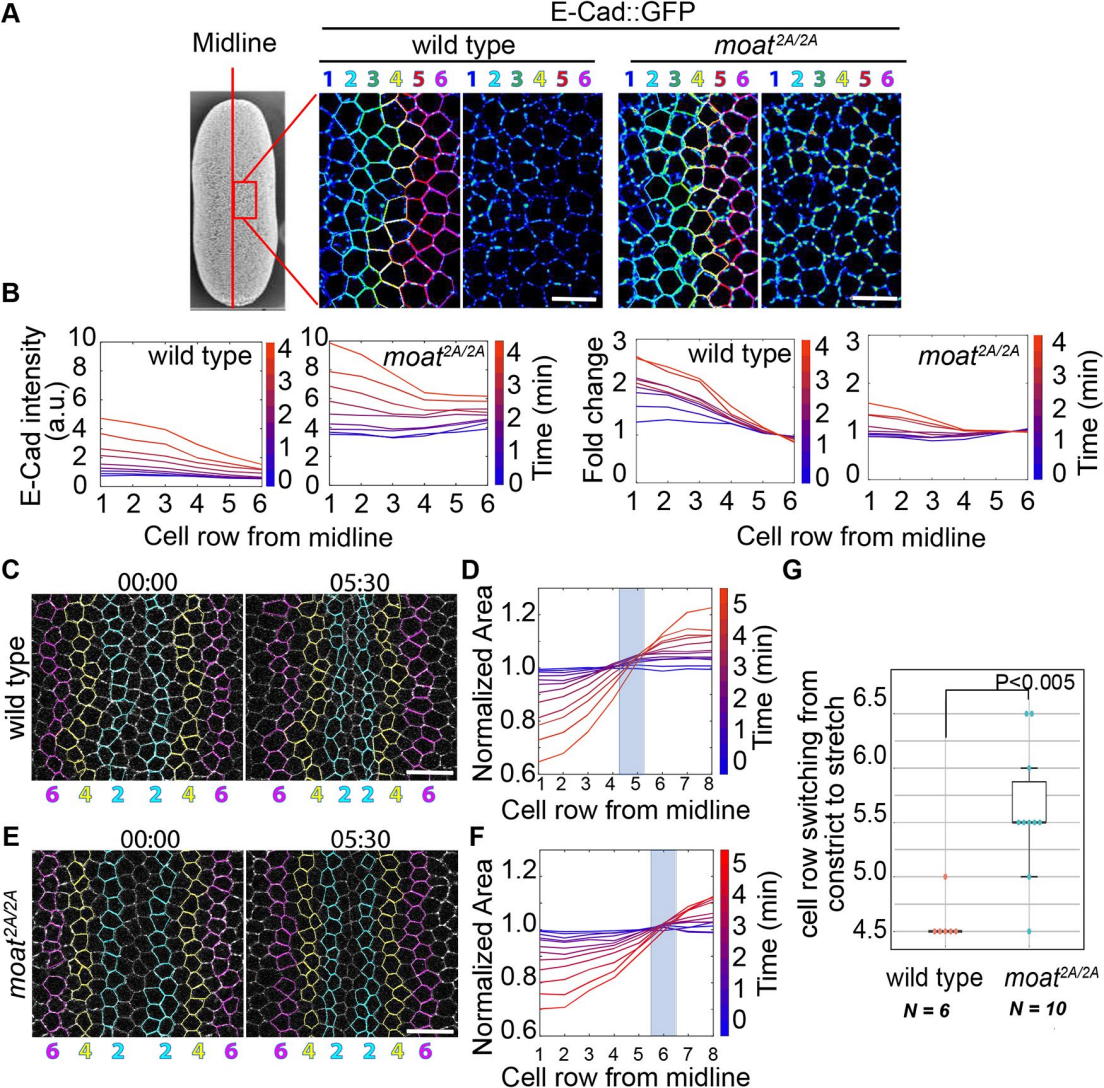
Ectopic apical constriction in *moat* mutant flanking mesoderm cells. (A) Individual cells were tracked and assigned row numbers based on their positions relative to the ventral midline. Heatmap images show relative E-Cad intensity. (B) Top panels: the intensity of spot adherens junctions of different cell rows over time. Lower panel: the relative intensity of spot adherens junctions normalized by row 5 and 6 over time. Each curve represents junction intensities across the rows from the middle to the side. The blue-to-red color spectrum represents timepoints. (C, E) Still images from time-lapse movies with tracked rows of cells on both sides of the midline. Numbers and colors are consistent with those in A. (D, F) Normalized apical area plotted against cell row number for the time-lapse movies in (C, E). Curves for individual timepoints are represented using the blue-to-red color spectrum. Each plot in B, D, and F shows measurements from individual embryos, and the number of cells in each row and at each timepoint may vary ranging from 23 to 32. (G) Quantification of the cell row number at which cells switch from constriction to expansion. Wild type: *N* = 6; *moat^2A/2A^*: *N* = 10. *p* = 0.0044 determined using Student’s *t* tests (two tails, two-sample unequal variance). Scale bar in A, C, and E: 10 µm. For C and E: T = mm:ss

To test whether loss of *moat* leads to ectopic apical constriction in the flanking mesoderm, we tracked rows of cells from the ventral midline to quantify changes in their apical areas (Figure 7A). E-Cad::GFP was used to label adherens junctions and the cell outline for tracking individual cells. In wild-type embryos, we observed a gradient of adherens junctions, stronger in cells closer to ventral midline (Figure 7B, left). This suggests a gradient of junctions is formed in response to the gradient of contractile myosin, consistent with the essential role of myosin-dependent junction mechanism (Weng and Wieschaus, 2016). This shows the wild-type flanking mesoderm cells resemble ectoAMG in both low contractile myosin and low adherens junctions. By contrast, in *moat* mutants, the flanking mesoderm shows significantly higher levels of adherens junctions due to high levels of junctional Baz, and the gradient of adherens junction levels becomes less pronounced (Figure 7B, right).

Wild-type mesoderm displays a highly reproducible gradient of apical constriction (Figure 7, C and D; Supplemental Movie S10). Cells in the first four rows from the midline show apical constriction while cells from row 5 start to show apical expansion (quantified in Figure 7D), in agreement with the previous report (Heer *et al.*, 2017). However, in *moat* mutant embryos, the cell row that starts apical expansion is shifted laterally to row 6 or 7 (Figure 7, E and F; Supplemental Movie S10). Meanwhile, the middle rows constrict but not as much as those in wild-type embryos. This indicates the flanking mesoderm cells ectopically undergo apical constriction, which may stretch the middle cells. We quantified the cell row that transitions from apical constriction to expansion. While the transition point occurs between rows 4 and 5 in wild-type embryos, it moves laterally to rows 5 and 6 in *moat* mutants (Figure 7G).

**Figure:** Movie S10

**FIGURE 8:**
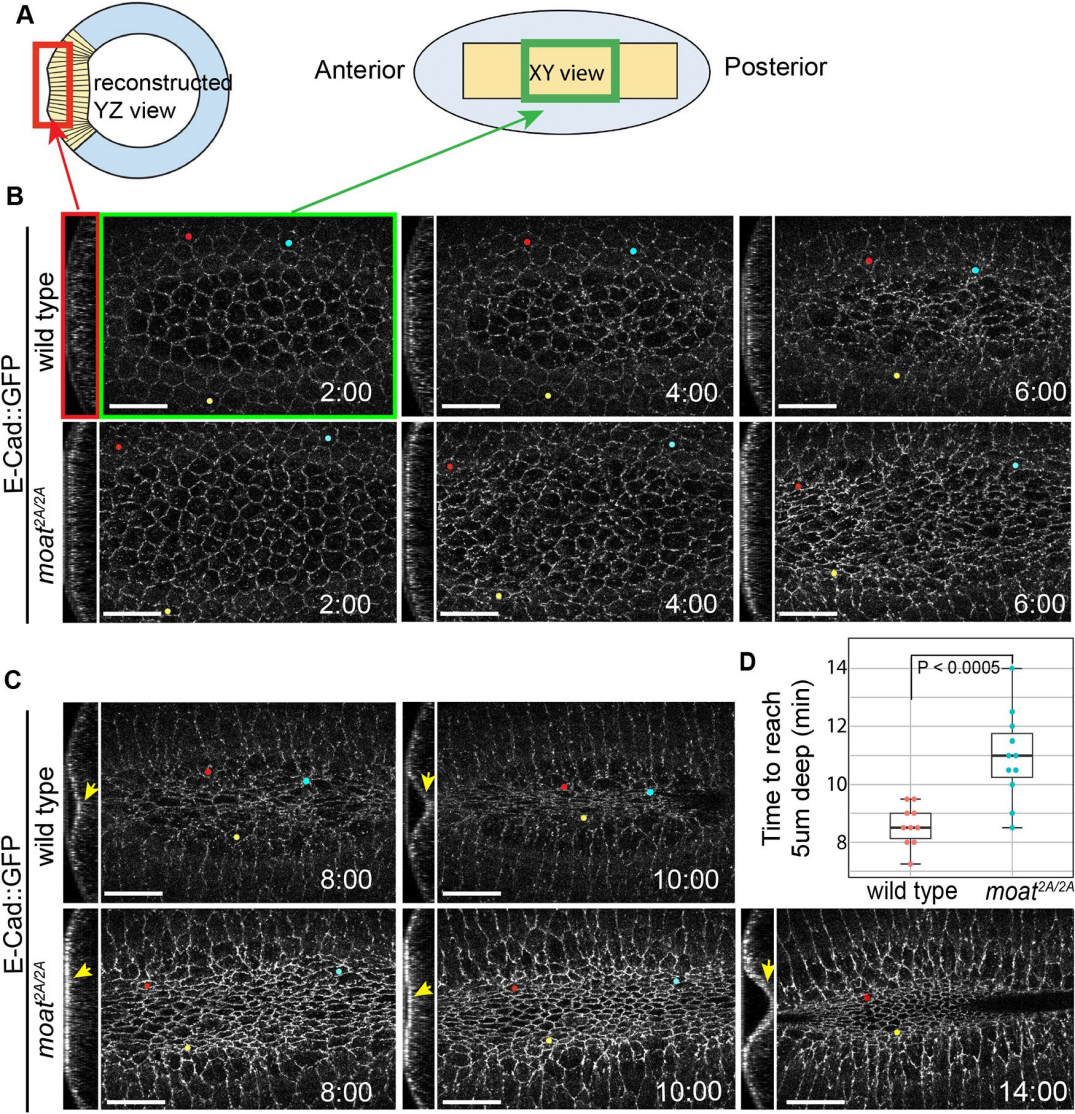
Epithelial folding is prolonged in *moat* mutant embryos. (A) Diagram illustrating the two views of constricting mesoderm shown in B and C. Left: cross section view (YZ) showing the depth of ventral furrow. Right: en face view (XY) showing the apical cell outlines. Mesoderm is shown in yellow. (B–C) Still images from time-lapse movies of folding mesoderm with YZ and XY views as illustrated in A. Tracked cells are marked with colored dots throughout the time. The YZ slices were obtained using the Reslice function in ImageJ. Scale bar in (A), (C), and (E): 20 µm. (D) Quantification of the time it takes for the apical surface of the furrow to reach 5 µm deep. Wild type: *N* = 10; *moat^2A/2A^*: *N* = 11. *p* = 0.00041 determined using Student’s *t* tests (two tails, two-sample unequal variance). For B and C: T = mm:ss

Because the gradient of apical constriction is essential for efficient ventral furrow formation (Heer *et al.*, 2017), we examined whether the disrupted gradient of constriction in *moat* mutants could reduce infolding efficiency. Initially, both wild-type and *moat* mutant mesoderm cells converge toward the ventral midline as the apical areas of the cells in the middle rows reduce, although the cells in the *moat* mutant embryo constrict more evenly across ventral-lateral axis (Figure 8, A and B; Supplemental Movie S11). After about 5 min, flanking mesoderm cells in wild-type embryos continue to move towards the midline as middle cells constrict even more, leading to the formation and deepening of the furrow (Figure 8C, upper panels, arrows showing depth; Supplemental Movie S11). Meanwhile, in *moat* mutant embryos, the cell movement toward the midline stalls and only a shallow furrow forms. It takes much longer for the furrow to reach a similar depth (Figure 8C, lower panels; Supplemental Movie S11). On average, *moat* mutant mesoderm takes 2.5 min longer than wild type to form a furrow 5 µm deep (Figure 8D). Thus, in *moat* mutant mesoderm, the lateral expansion of apical constriction results in less coordinated and prolonged infolding. Taken together, our data argue the downregulation of junctional Baz in wild-type ventral furrow may ensure an optimal gradient of apical constriction and therefore robust internalization of mesoderm.

**Figure:** Movie S11

## DISCUSSION

The engagement between contractile myosin and adherens junctions executes cell shape changes during many morphogenetic events such as epithelial folding. While it has been shown that a multicellular gradient of contractile myosin determines the curvature of infolding epithelium (Heer *et al.*, 2017), how the tissue-scale distribution of cell adhesion determines the differential cell shape changes across the tissue is not well understood. Here, we used the various tissues within the Sna-expressing zone of fly early embryo to investigate morphogenetic boundaries. To identify the boundaries, we clarified the tissue segments within Sna-expressing zone from anterior to posterior: 1) Hkb(+) Gt(−) ectoAMG that participates in neither ventral furrow nor stomodeum invagination; 2) the uncharacterized Hkb(−) Gt(+) cells that forms the anterior end of ventral furrow; 3) endoAMG, and 4) mesoderm, both Hkb(−) Gt(−) and forming the rest of the ventral furrow (Figure 2B). In addition, we found that stomodeum and ectoAMG are two separate invagination events and it is more appropriate to define stomodeum as the cells anterior to ectoAMG rather than including ectoAMG.

With this system, we identified a novel Drosophila gene *moat* that modulates the tissue-specific distribution of adherens junctions through limiting the junctional localization of polarity protein Baz. This Moat-dependent regulation of junction levels is essential for preventing ectopic apical constriction in cells with low-level contractile myosin, thereby maintaining the normal morphogenetic boundaries. We found that in *moat* mutant embryos: along the anterior-posterior axis, the infolding behavior of ventral furrow is expanded to ectoAMG, and along the ventral-lateral axis, the strong apical constriction behavior of middle mesoderm cells is expanded to the flanking mesoderm cells.

**FIGURE 9:**
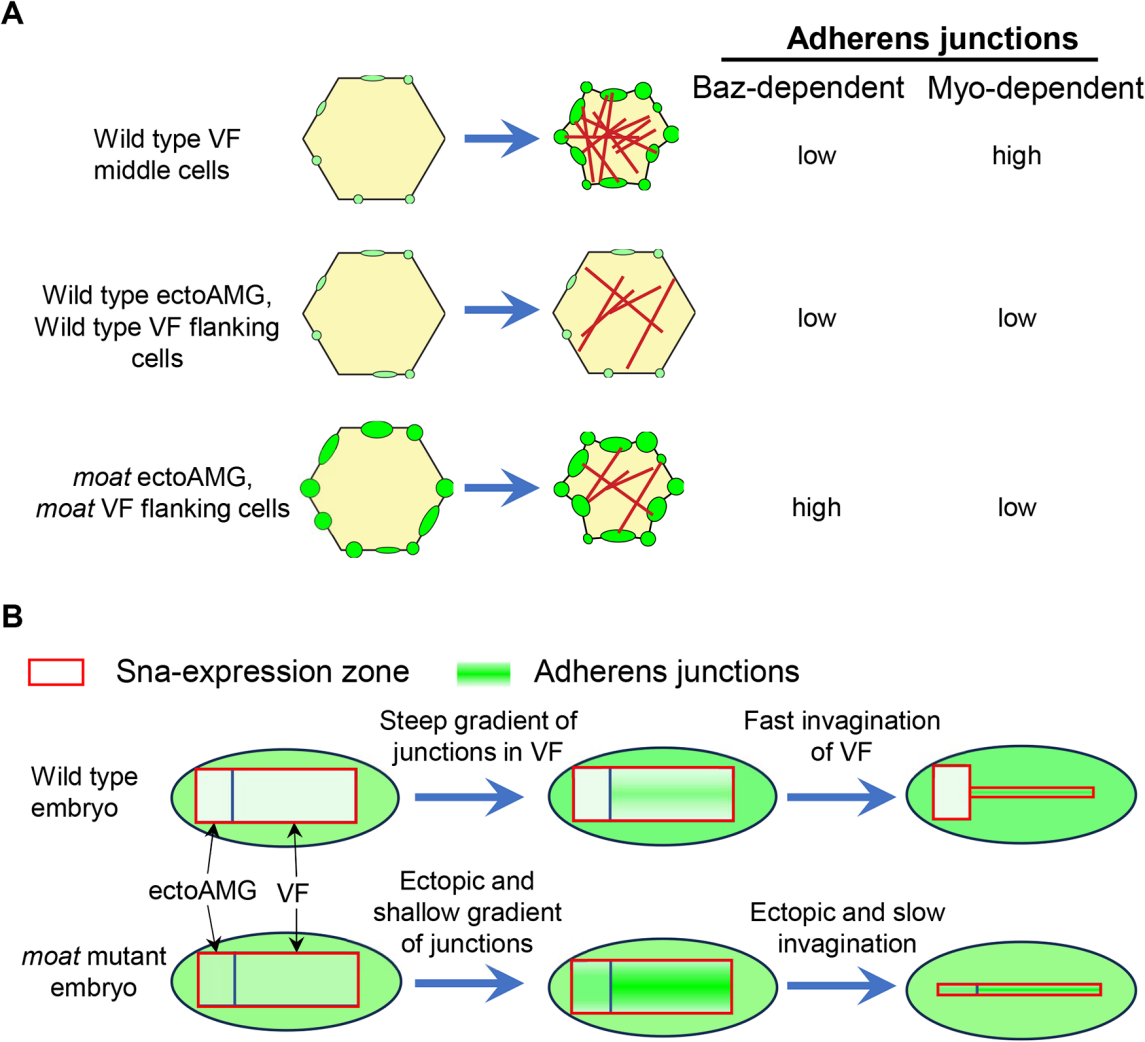
Model for how the levels of myosin and adherens junctions determine apical constriction outcome. (A) The levels of both myosin and adherens junctions determine the outcome of apical constriction. In cells where Baz-dependent junctions are downregulated, effective apical constriction relies on sufficient levels of myosin-dependent junctions and cannot occur when myosin levels are low. However, cells with low levels of myosin can undergo ectopic constriction in *moat* mutants due to elevated levels of junctional Baz. (B) At tissue levels, downregulation of Baz-dependent adherens junctions in Sna-expression zone allows the formation of a gradient of myosin-dependent junctions in ventral furrow region, leading to coordinated fast folding of ventral furrow excluding ectoAMG. In *moat* mutant embryos, Baz-dependent junctions persist in the entire Sna-expression zone. This leads to ectopic apical constriction in ectoAMG and flanking mesoderm cells, resulting in ectopic folding of ectoAMG and inefficient folding of ventral furrow, respectively.

Our results propose a model where the combination of different levels of contractile myosin and adherens junctions determines the levels of apical constriction and the subsequent cell shape changes (Figure 9). Our results suggest that the landscape of adherens junction levels is shaped in a two-step mechanism. First, the Baz-dependent adherens junctions uniformly distributed across the embryo are specifically downregulated in the entire Sna-expressing zone. Next, with the clearance of Baz-dependent adherens junctions, a new distribution of adherens junctions arises in this zone in response to the gradient of contractile myosin: a gradient of adherens junctions forms specifically in ventral furrow cells, whereas junction levels in ectoAMG remain to be low. The gradients of contractile myosin and adherens junctions together lead to a sharpened gradient of apical constriction and robust infolding of ventral furrow. Whereas, the low levels of both contractile myosin and adherens junctions exclude ectoAMG from ventral furrow infolding. In *moat* mutant ectoAMG and flanking ventral furrow cells, the low-level contractile myosin combined with the abnormally high levels of Baz-dependent adherens junctions pushes the level of apical constriction above the threshold for epithelial folding. We showed that Baz is essential for the ectopic folding in *moat* mutant ectoAMG. This is distinct from normal ventral furrow, where Baz-dependent junctions are less critical since the strong contractile myosin induces high levels of myosin-dependent junctions.

Past studies of Sna-dependent Baz downregulation raise the question of what is the benefit of weakening junctions before apical constriction, a process that requires strong junctions. Our model suggests the clearance of Baz-dependent adherens junctions in the entire Sna-expressing zone is essential in reestablishing a new landscape of adherens junctions that matches that of contractile myosin. In ectoAMG, this reduces the chance of ectoAMG undergoing ventral furrow-like infolding, especially when the suppression of contractile myosin by Hkb is incomplete. In mesoderm cells, this maximizes the gradient of apical constriction and increases the folding efficiency. Moat could represent the module that optimizes the levels of adherens junctions in the scheme of fast epithelial folding. *moat*, along with genes such as *fog*, *T48,* and *fruhstart* that specifically function in ventral furrow cells at gastrulation stage, ensure successful infolding of mesoderm (Urbansky *et al.*, 2016). They provide us opportunities to understand the shaping of epithelial folds.

Moat appears to facilitate Sna in downregulating junctional Baz by reducing the junctional localization of Baz. The junctional puncta of Baz appear to be bigger and more intense in the *moat* mutants, suggesting Moat may limit the size and density of Baz puncta at junctional cortex. The condensation of Baz proteins into punctate structures at the cortex has been shown to be driven by liquid-liquid phase separation (Liu *et al.*, 2020). Many mechanisms collectively contribute to such puncta formation, including the oligomerization and membrane association of Baz proteins, interaction of Baz with other cortical polarity proteins, and microtubule-dependent transport of Baz puncta (Harris, 2017). Our data using endogenously tagged Moat show that Moat associates with the cell membrane or cortex (Supplemental Figure S1C). This raises the possibility that Moat may interact with cortically localized Baz or its regulators, thereby reducing the interactions required for puncta formation at the junctional cortex. Alternatively, Moat may change the local membrane composition and make the junctional cortex less favorable for Baz to form puncta.

What is the molecular and cellular mechanism by which adherens junction levels enhance apical constriction? One possibility is through increasing the effective interaction between actomyosin filaments and cell cortex. Not all contractile actomyosin filaments in the cell are engaged with adherens junctions to constrict apical cortex. An increase in the number of junction puncta and the amount of Cadherin–Catenin complexes per puncta could increase the frequency of actomyosin filament engagement. Another possibility is that high levels of adherens junctions may themselves function as a platform to increase actomyosin assembly or recruitment of myosin activators. Answering this question will shed light on the mechanism of adherens junctions and myosin interactions.

## MATERIALS AND METHODS

Request a protocol through *Bio-protocol*.

### Fly stocks

Fly lines are listed in Supplemental Table S1

### Generation of fly strains

*moat* mutant allele was generated using CRISPR technique. Two guide RNA fragments were selected: one in the 5′-UTR (untranslated region) close to the start codon (gRNA-5) and the other in the exon close to the stop codon (gRNA-3). The phosphorylated gRNA oligos were annealed and cloned into pU6-BbsI-ChiRNA vector (flycrispr.org). For the donor template construct, a 901 bp 5′ homology arm and a 1017 bp 3′ homology arm were amplified and cloned into a pHD-DsRed vector 5′ and 3′ MCS via enzyme restriction, respectively. The oligonucleotides for gRNA and primers for donor constructs are listed below:

gRNA-5 sequence: 5′-CATGCGGTACGGGTGATTCC**AGG**-3′
 Sense oligo: 5′-cttcGCATGCGGTACGGGTGATTCC-3′
   Antisense oligo:5′- aaacGGAATCACCCGTACCGCATGC-3′
gRNA-3 sequence: 5′-GCGTGTGCCCGGCTAGCAAT**TGG**-3′
 Sense oligo:5′-cttcGCGTGTGCCCGGCTAGCAAT-3′
   Antisense oligo:5′-aaacATTGCTAGCCGGGCACACGC-3′
5′ homology arm: F: 5′-TGCACA*gaattc*TGAGATCCCACGTG­TTCGTC-3′ (*EcoRI*)
 R: 5′-TGCACA*cggccg*GCATGCGGCTACCTGATTAGT-3′ (*EagI*)
3′ homology arm: F: 5′-TGCAGC*actagt*AATTGCTAGCCGGGCACAC-3′ (*Spel*)
 R: 5′-TGCAGC*ctcgag*CGCAATCAGTTAATGGGCTGG-3′ (*Xhol*)

The donor and two gRNA constructs were injected into w*; FRT40A; nos-Cas9 attP2 strain by Rainbow Transgenic Flies. The flies carrying the deletion were confirmed by dsRed fluorescence in fly eyes and by PCR using adult fly genomic DNA as a template. The primers for PCR: F: 5′-CACGGCTAGACGGCATTTCT-3′ and R: 5′-GCAGCTCATGAAAGTCAGGC-3′.

To generate moat rescue flies, the bacterial artificial chromosome (BAC) carrying a small genomic DNA fragment covering *moat* and *nullo* (CH322-67O8) was obtained from the BACPAC Resources Center (BPRC). Embryo injection was done by the BestGene using y^1^w67^c23^; p{CaryP}attP40 strain.

### Embryo fixation and immunostaining

Drosophila embryos were collected on apple juice agar plates at 25°C for 2 h, followed by an additional 2-h aging period at the same temperature after the removal of adult flies. Then the embryos were dechorionated with 50% of 8% household bleach (4% sodium hypochlorite) and fixed by heat-methanol protocol as described before (Müller and Wieschaus, 1996). Briefly, in a 15 ml glass vial, the dechorionated embryos were incubated in 3 ml of hot salt solution (0.4% NaCl, 0.03% Triton x-100) for 10 s, and then swiftly cooled by adding 2 volumes of prechilled salt solution. Discarded the salt solution from the vial and then added a 1:1 mixture of methanol and heptane. Vortexed vigorously for at least 30 s to remove the vitelline membranes. Then, the embryos were transferred into an Eppendorf tube washed with methanol three times. The embryos were stored in methanol at −20°C until use.

Embryos were incubated in 10% BSA blocking buffer for 1 h, followed by overnight staining with primary antibody at 4°C and subsequent staining with the secondary antibody for 2 h at room temperature. The primary and secondary antibodies used in this study are listed in Supplemental Table S2. Following antibody staining, embryos were sorted and mounted in Aqua-PolyMount (Polysciences). Images are acquired on a Zeiss LSM800 confocal microscope equipped with high-sensitive GaAsp detectors. LD LCI plant-apochromat 25x/0.8 objective was used for whole embryo images and plan-Apochromat 63x/1.4 NA Oil DIC M27 objective was used for high magnification images.

### Live imaging

All images were acquired on the Zeiss LSM800 confocal microscope described above. An LD LCI plant-apochromat 25x/0.8 NA oil objective was used for the anterior midgut imaging and Baz quantification. A Plan-Apochromat 63x/1.4 NA Oil DIC M27 objective was used for E-Cad quantification and mesoderm cell tracking. Diode 488 and 561 nm lasers were used to excite GFP and mCherry, respectively. The pinhole is set at 1 Airy unit for 488 for all images. Zeiss Definite Focus was used to maintain the focal plane.

For mesoderm regions, embryos were prepared using the protocol as described (Gu and Weng, 2021). To quantify Baz and E-Cad in the mesoderm (Figure 5), images were captured at a lower temporal and spatial resolution to minimize photobleaching: a temporal resolution of 1 min per z-stack, a z step of 1 µm and a xy resolution of 0.25 µm and 0.2 µm per pixel, respectively. Baz high magnification images were obtained at a temporal resolution of 30 s per z-stack, a z step of 0.5 µm, and an xy resolution of 0.13 µm per pixel.

For the anterior midgut, embryos were mounted and imaged differently in order to capture most of the ectoAMG on the highly curved anterior end of the embryo with high quality. Embryos were tilted along the anterior-posterior direction during mounting so that the anterior quarter of the embryo landed on the cover glass. Images were acquired with a xy resolution of 0.25 µm per pixel, a z-step of 1 µm, a total depth of 37 µm, and a temporal resolution of 30 s per z-stack.

For cell tracking and apical area measurements, images were captured at a xy resolution of 0.2 µm per pixel, a z-step of 1 µm, a total depth of 18 µm, and a temporal resolution of 30 s per z-stack.

### SEM

Embryos were dechorionated with 50% of 8% household bleach (4% sodium hypochlorite) and fixed for 25 min with 25% glutaraldehyde in 0.1 M sodium cacodylic buffer and heptane. The vitelline membrane was then manually removed in PBS with a needle, and embryos were dehydrated by a gradient of ethanol concentration (25%, 50%, 75%, 95%, and 100%). Embryos were then incubated for 10 min in a 1:1 mixture of ethanol and hexamethyldisilazane (HMDS), followed by two additional incubations with 100% HMDS. After HMDS evaporated completely, embryos were transferred to the SEM stub and gold coated using a Sputter Coater 108auto (Cressington). Samples were imaged using Hitachi TM-1000.

### Imaging processing and analysis

All images for publication were processed in ImageJ (http://rsb.info.nih.gov/ij/). Brightness and contrast were adjusted for the whole image. Quantitative analysis was done using MATLAB (MathWorks).

To quantify the spot adherens junctions and junctional Baz (Figure 5), a region of about 9 × 15 mesoderm cells was used for quantification. Images were thresholded to exclude the uniform and weak membrane or cytoplasmic signals. The threshold is based on the z section 7 µm from the embryo surface, where there are little junctional Baz or spot adherens junctions. The threshold is calculated as pixel value mean plus 7 SDs for E-Cad::GFP images and mean plus 3 SDs for Baz::GFP images. The difference in the threshold calculation is mainly due to the difference in the distribution of the two proteins outside the puncta pool. While nonpuncta E-Cadherin diffuses in the membrane with little cytoplasmic localization, nonpuncta Baz is evenly distributed in the cytoplasm with no membrane localization. This leads to much higher nonpuncta Baz levels. The mask generated from the initial thresholding is further processed to remove objects (groups of connected pixels) smaller than 4 pixels to generate the final mask for quantification (Supplemental Figure S4A). The intensity curve was normalized by the mean intensity of different samples at the starting timepoint.

For ventral apical area tracking (Figure 7), live imaging stacks were first flattened to correct the cell tilting caused by embryo curvature using HWada plugin tool in ImageJ (Wada and Hayashi 2020). The flattened images were used for cell tracking performed by a MATLAB package, Embryo Development Geometry Explorer (EDGE) (Gelbart *et al.*, 2012). EDGE detects the *xy* coordinates of cell vertices which are used for segmenting and tracking cells. Cell segmentation errors were manually corrected. The section at 4 µm deep from the apical surface was used to calculate apical area because the severe deformation of cell outline in the more apical sections prevents accurate segmentation and tracking. To group cells in rows, we defined the middle two cell rows using the timepoints when the furrow was a few microns deep and traced the cell rows back to early timepoints. We plotted the average apical area of all cells in each row over time. The apical areas of individual rows are normalized by the areas of corresponding row at the starting timepoint to show the fold change.

The intensity of spot adherens junctions in Figure 7 was measured in a similar way as that in Figure 1. The cell rows were defined from the cell tracking. To quantify E-Cad accurately, embryos that were mounted slightly off-center relative to the ventral midline were used for quantification and only six rows on one side of the embryo were plotted. This is because the fluorescence from the cells at the position that directly touch the coverslip is less refracted and artificially stronger than that from the cells positioned on the flanking slopes of the embryo. The gap between the slope of the embryo and the coverslip leads to loss of signals.

### Quantification of embryo phenotype

The fixed embryos, stained with Nrt or Arm, or imaged by SEM, were manually categorized based on their phenotype.

For scoring ectopic ectoAMG furrow phenotype, embryos of late stage 6 and stage 7 were used. Embryos with putative ectoAMG cells exhibiting relaxed apical surfaces that follow the curvature of the embryos were classified as having a normal end. Embryos with putative ectoAMG exhibiting apical constriction and forming a furrow were defined as having an extended end. (Figures 1C and 6C)

For scoring ventral furrow closure phenotypes (Figure 6D), embryos were sorted by whether the middle 1/3 of the ventral furrow is closed. Embryos from late stage 6 and stage 7 were used for quantification. Ventral furrows in wild-type embryos usually close by late stage 6 and become less distinguishable by stage 8 when domain 14 enters cell cycle.

### Western blot

Drosophila embryos were collected on apple juice agar plates at 25°C for 2 h, followed by an additional 2-h aging period at the same temperature after the removal of adult flies. Then the embryos were dechorionated by 50% of 8% household bleach (4% sodium hypochlorite). Embryo extraction was conducted by incubating dechorionated embryos in 2x Laemmli buffer (Bio-Rad) with 5% 2-mercaptoethanol at 95°C for 2 min. The lysates were then cleared by centrifugation at 16,000 × *g* for 5 min. 8% surePAGE gels (GenScript) were used for electrophoresis and Immobilon-FL polyvinylidene fluoride (PVDF) membranes (Millipore) were used for transfer. Membranes were blocked for 1 h in PBST (0.1% Tween-20 in PBS) containing 10% dry milk. Then the membranes were incubated in primary antibody overnight at 4°C (Guinea pig anti-Baz, 1:500; Mouse anti-Tubulin, 1:2000) and subsequently in secondary antibody for 1 h at room temperature (IRDye-coupled secondary antibody: Donkey anti-Gp 800CW catalog no. 926-32411 and Goat anti-Mouse 680RD catalog no. 926-68070, 1:5000). Membranes were visualized with an Odyssey infrared imaging system (LI-COR Bioscience), and brightness and contrast were adjusted for the whole image using ImageStudio software.

### mRNA in situ hybridization

The probe template was generated using the following primers: Forward: 5′-TGCAGAACAGTCGACAAGGA-3′ and Reverse: 5′-GCTGGGCAATGCAATCGTAGC-3′. DIG-labeled antisense and sense probes were synthesized using T7 and SP6 RNA polymerase following the instructions provided in the MGEA script kit from Applied Bio. For hybridization, the eggs were dechorionated in 4% sodium hypochlorite for 1 min, fixed for 20 min in a 1:1 mixture of heptane and 4% paraformaldehyde in PBS, and devitellinized in 1:1 methanol/ heptane mixture and washed in PBST. In situ hybridizations on whole-mount fixed eggs were essentially performed as described before (Tautz and Pfeifle, 1989). Then the embryos were blocked and incubated with primary antibody (anti-DIG-AP, Millipore Sigma catalog no. 11093274910) for 2 h at room temperature. For color development reaction, the embryos were incubated in NBT/BCIP solution (Millipore Sigma, catalog no. 11681451001) at room temperature in the dark. The reaction was stopped by washing several times in PBS with 0.1% Tween 20. Embryos were photographed using a Nikon 90i Eclipse microscope with S Fluor 10x DIC N1 optics and a motorized stage.

### Statistical analysis

Statistical analyses in Figures 7 and 8 were conducted using a two-tailed unequal Student’s *t* test.

## Supplementary Material



## Abbreviations used:

Arm: Armadillo
Baz: Bazooka
α-Cat: α-Catenin
E-Cad: E-Cadherin
ectoAMG: ectodermal anterior midgut
endoAMG: endodermal anterior midgut
Gt: *giant*
Hkb: huckebein
SEM: scanning electron microscopy
Sna: Snail
VF: ventral furrow
