# Supplementary material for: A novel protein Moat prevents ectopic epithelial folding by limiting Bazooka/Par3-dependent adherens junctions
Source: Mol Biol Cell. 2024 Jul 22;35(8):ar110. doi: 10.1091/mbc.E24-04-0177 (PMC11321041; doi:10.1091/mbc.E24-04-0177)
Supplement: Supplementary file 12 [file mbc-35-ar110-s001.pdf]

Supplemental Materials

Molecular Biology of the Cell

Gu *et al*.

Table S1. Fly stains used in this study:

Fly strain	Sources and References
w1118(wild type)	
moat$^{2A/2A}$	this study
Baz::GFP[1] (CRISPR C-terminus tag)	this study
ubi-EcadGFP	Oda and Tsukita, 2001
sqh::mCherry	Martin et al., 2009
Moat$^{p[acman]}$	this study
cat$^{R10/R10}$*/CyO;T48*$^{cc1.2/cc1.2}$	Kolsch et al., 2007
w;67;15	Hacker and Perrimon, 1998
uas-aCatRNAi	BDSC #33430
uas-luciferase RNAi	BDSC #31603
uas-Baz RNAi(II)	BDSC #39072
uas-Baz RNAi(III)	BDSC #35002

1. **Häcker, U. and Perrimon, N.** (1998). DRhoGEF2 encodes a member of the Dbl family of oncogenes and controls cell shape changes during gastrulation in Drosophila. Genes Dev 12,.

2. **Kölsch, V., Seher, T., Fernandez-Ballester, G. J., Serrano, L. and Leptin, M.** (2007). Control of drosophila gastrulation by apical localization of adherens junctions and RhoGEF2. Science (1979) 315,.

3. **Martin, A. C., Kaschube, M. and Wieschaus, E. F.** (2009). Pulsed contractions of an actin-myosin network drive apical constriction. Nature 457,.

4. **Oda, H. and Tsukita, S.** (2001). Real-time imaging of cell-cell adherens junctions reveals that Drosophila mesoderm invagination begins with two phases of apical construction of cells. J Cell Sci 114,.

Table S2. Antibodies used in this study:

Antibody	Host Species	Source	Dilution
anti-Arm	Mouse	DSHB, AB_528089	1:50
anti-Neurotactin	Mouse	DSHB, AB_528404	1:200
anti-Bazooka	Rabbit	This study, using the Baz construct from Wodarz Lab[1]	1:500
anti-Bazooka	Guinea Pig	This study, using the Baz construct from Wodarz Lab[1]	1:200
anti-Snail	Rat	Wieschaus Lab (Princeton University)	1:1000
anti-Snail	Guinea Pig	Wieschaus Lab (Princeton University)	1:1000
anti-Giant	Guinea Pig	Wieschaus Lab (Princeton University)	1:1000
anti-Giant	Rat	Wieschaus Lab (Princeton University)	1:500
anti-Huckebein	Rat	Doe Lab[2] (University of Oregon)	1:100
Alexa Fluor 488	Goat or Donkey	Invitrogen	1:500
Alexa Fluor 568	Goat or Donkey	Invitrogen	1:500
Alexa Fluor 647	Goat or Donkey	Invitrogen	1:500
IRDye 680RD anti-mouse	Goat	LI-COR	1:5000
IRDye 800CW anti-Guinea Pig	Donkey	LI-COR	1:5000

1. **Wodarz, A., Ramrath, A., Kuchinke, U. and Knust, E.** (1999). Bazooka provides an apical cue for inscuteable localization in Drosophila neuroblasts. *Nature* **402**,.

2. **McDonald, J. A. and Doe, C. Q.** (1997). Establishing neuroblast-specific gene expression in the Drosophila CNS: huckebein is activated by Wingless and Hedgehog and repressed by Engrailed and Gooseberry. *Development* **124**,.

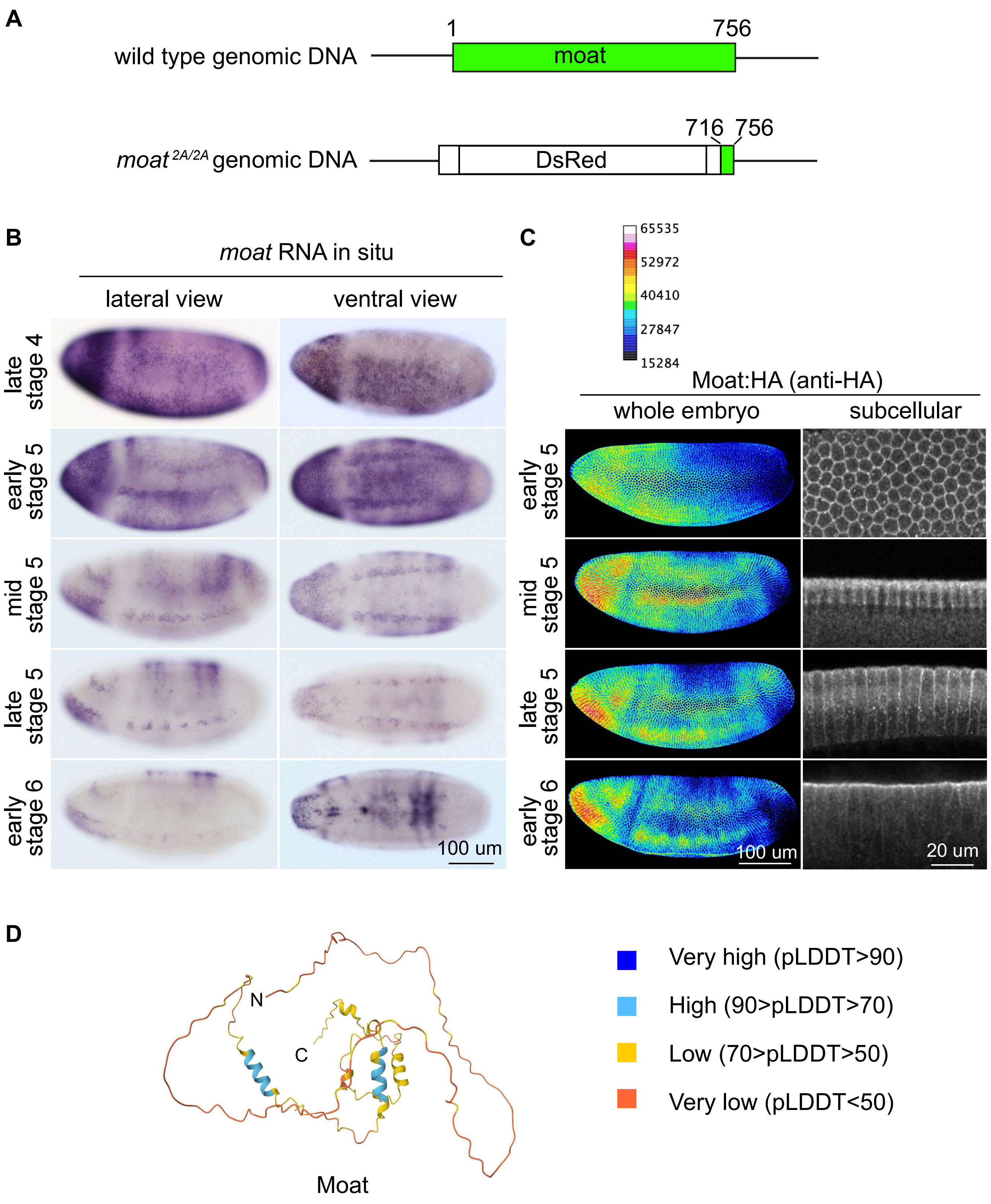
A
1
756
wild type genomic DNA
moat
716
756
moat 2A/2A genomic DNA
DsRed
B
moat RNA in situ
lateral view
ventral view
late stage 4
early stage 5
mid stage 5
late stage 5
early stage 6
100 um
C
65535
52972
40410
27847
15284
Moat:HA (anti-HA)
whole embryo
subcellular
early stage 5
mid stage 5
late stage 5
early stage 6
100 um
20 um
D
N
C
Moat
Very high (pLDDT>90)
High (90>pLDDT>70)
Low (70>pLDDT>50)
Very low (pLDDT<50)

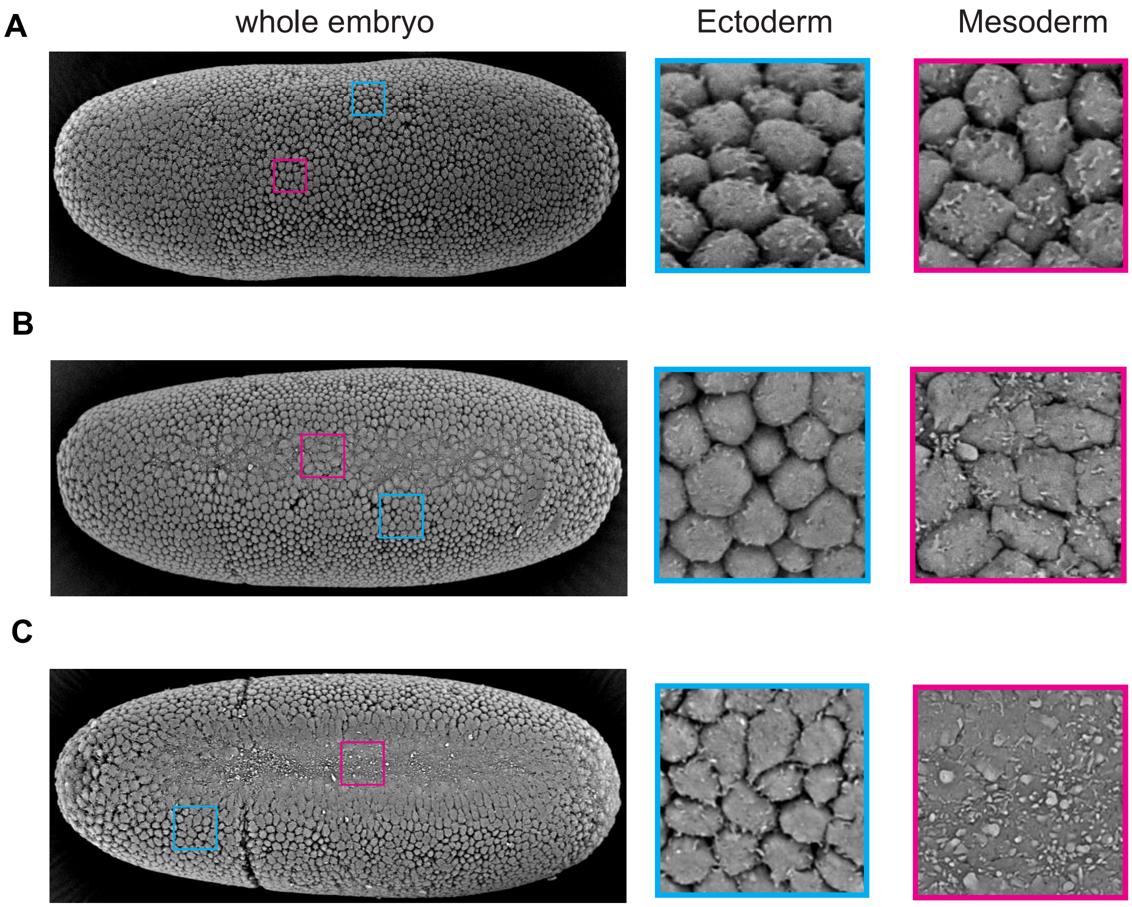
A
whole embryo
Ectoderm
Mesoderm
B
C

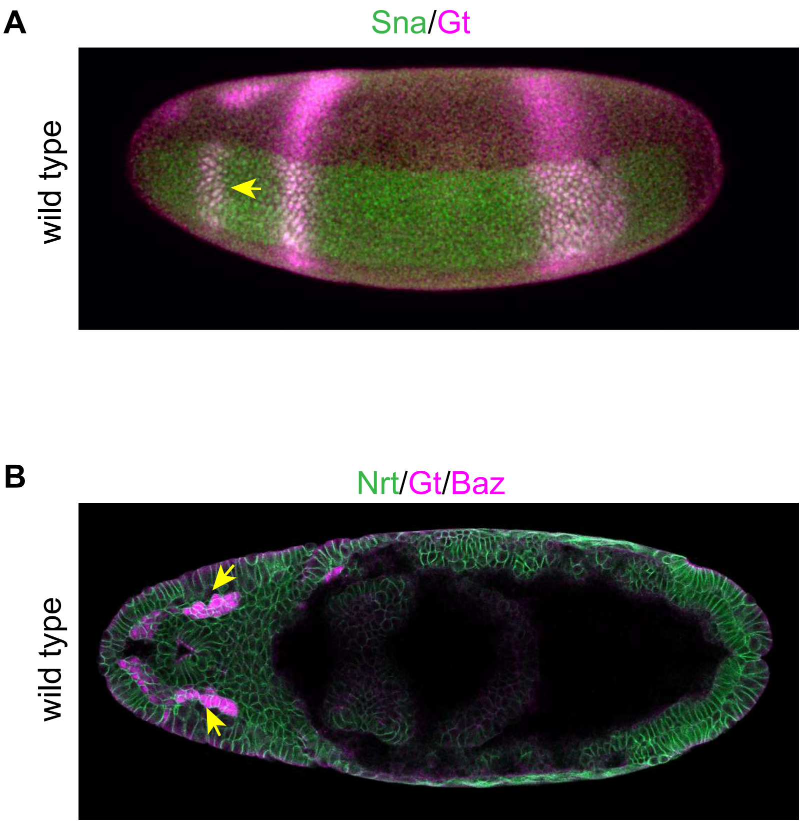
A
Sna/Gt
wild type
B
Nrt/Gt/Baz
wild type

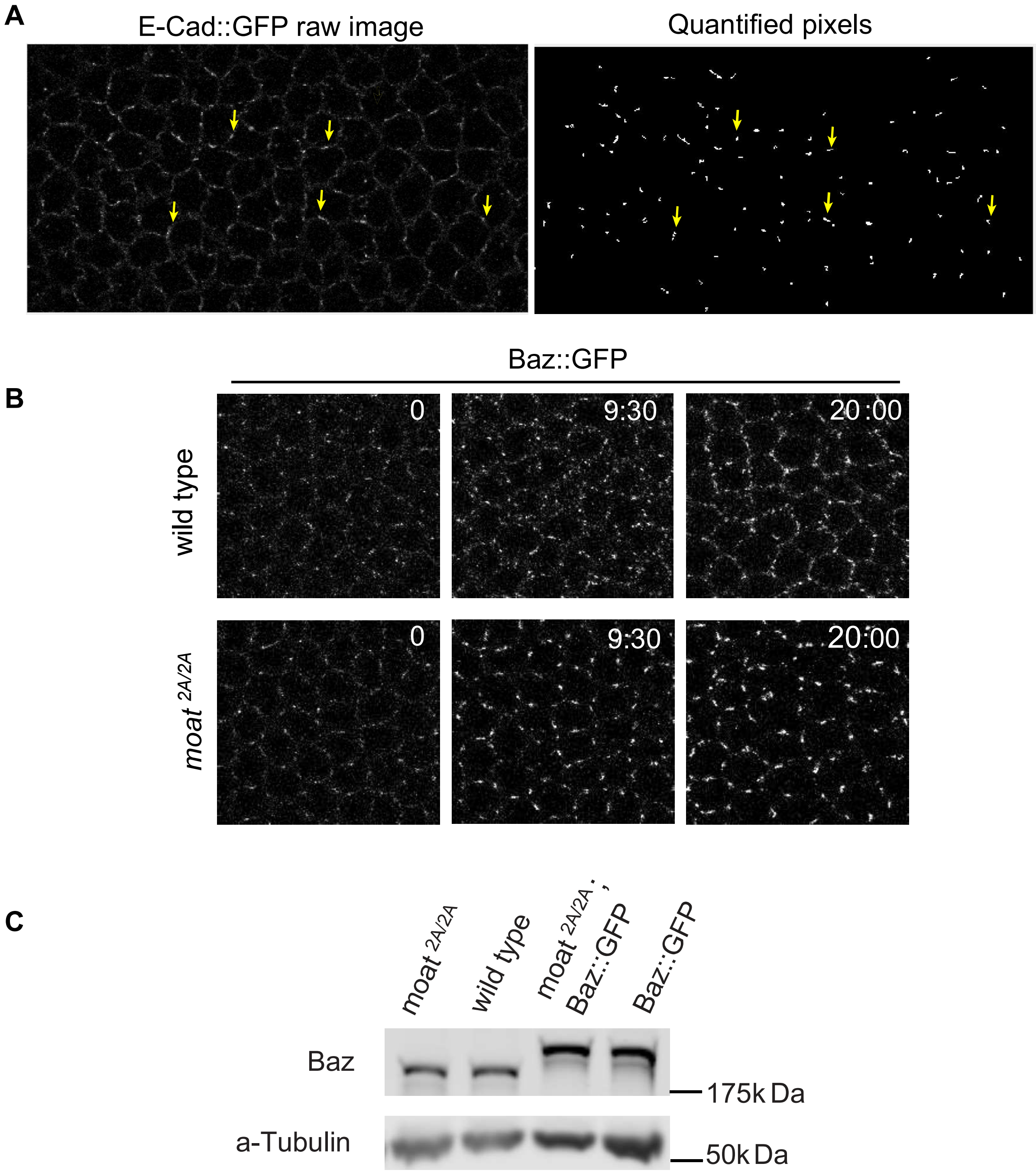

A
E-Cad::GFP raw image
Quantified pixels
B
Baz::GFP
wild type
0
9:30
20:00
moat 2A/2A
0
9:30
20:00
C
moat 2A/2A
wild type
moat 2A/2A ; Baz::GFP
Baz::GFP
Baz
175k Da
a-Tubulin
50k Da

Figure S1. *moat* shows dynamic expression patterns during early embryogenesis.

(A) Schematic representation of *moat* gene region in wild type and CRISPER/Cas9 knockout flies. (B) In situ hybridization for *moat* mRNA in wild-type embryos from stage 4 to early stage 6 showing both lateral and ventral views of the embryos. (C) Expression patterns of HA-tagged Moat protein revealed by immunostaining against HA in whole embryos from stage 5 to early stage 6. Left panel: whole embryos with HA signals in a heatmap; Right panel: Subcellular distribution of Moat protein in mesoderm cells in corresponding stages. Early stage 5: en face view; Mid-stage 5 to early stage 6: z-sections along apical/ basal axis. (D) AlphaFold prediction of Moat protein structure. pLDDT: per-residue model confidence score.

Figure S2. Giant protein expression pattern in wild type embryos.

(A) Ventrolateral max projection of a cellular blastoderm wild type embryo immunostained for Gt and Sna. Yellow arrow: ventral anterior Gt stripe. (B) Single optical section of a stage 10 wild type embryos immunostained for Nrt, Gt and Baz. Yellow arrows: Gt(+) cells derived from the Gt(+) ventral strip.

Figure S3. Cell shape changes in response to myosin activity.

(A-C) Scanning electron microscopy (SEM) images of wild type embryos of stages before myosin activation, at the beginning of myosin activation, and when myosin is strongly activated. Left panel: whole embryos. Right panel: high magnification images of boxed regions in whole embryos. Cyan boxes: lateral ectoderm region; Magenta boxes: mesoderm region

Figure S4. The recruitment of Baz clusters was altered in *moat* mutant embryos.

(A) The mask generated for junctional Baz quantification. Left: Original image; Right: the binary image that shows the pixels (mask) used for quantification. Yellow arrows: pointing to the Baz puncta in the original image and their corresponding pixel groups in the mask. (B) Still images from a time-lapse movie of Baz::GFP in the ectoderm of wild type and moat mutant embryos shortly before apical constriction. T=mm:ss （C） Western blot of Baz protein in wild type and moat mutant embryos. α-tubulin served as the loading control.
