# Supplementary material for: A novel protein Moat prevents ectopic epithelial folding by limiting Bazooka/Par3-dependent adherens junctions
Source: Mol Biol Cell. 2024 Jul 22;35(8):ar110. doi: 10.1091/mbc.E24-04-0177 (PMC11321041; doi:10.1091/mbc.E24-04-0177)
Supplement: Supplementary file 13 [file reviewer_reportE24-04-0177.pdf]

A novel protein Moat prevents ectopic epithelial folding by limiting Bazooka/Par3-dependent adherens junctions

Lingkun Gu, Rolin Sauceda, Jasneet Brar, Ferdos Fessahaye, Minsang Joo, Joan Lee, Jacqueline Nguyen, Marissa Teng, and Mo weng

Corresponding author(s): Mo weng, University of Nevada, las vegas

Review Timeline:

Submission Date:	2024-04-23
Editorial Decision:	2024-05-20
Revision Received:	2024-06-12
Accepted:	2024-06-13

Editor-in-Chief: Matthew Welch

Transaction Report:

(Note: With the exception of the correction of typographical or spelling errors that could be a source of ambiguity, letters and reports are not edited. The original formatting of letters and referee reports may not be reflected in this compilation.)

RE: Manuscript #E24-04-0177
TITLE: "A novel protein Moat prevents ectopic epithelial folding by limiting Bazooka/Par3-dependent adherens junctions"

Dear Prof. weng:

I'm pleased to say that two reviewers feel this work is suitable for MBoC and I agree with that assessment. I believe the paper can be brought into publishable form with text revision only.

That said, both reviewers do ask that you spend some time revising the manuscript and adding quite a few additional details. I'd ask you to address their requests for text edits to the extent possible. Reviewer #1's questions about the Moat protein seem especially important to me.

You will see that Reviewer #2 has asked for an additional experiment to test the idea that the phenotype may be related to ectopic sites of furrowing. I do not agree that additional experiments are needed and I and will not require it. I do, however, agree that this possibility should be explicitly addressed in your revised manuscript. (I note as well that if you have data on this point, or you wish to do this experiment and add it to the paper, that is of course acceptable. Just know that this is not a requirement for publication.)

I look forward to seeing your revised version published in MBoC. Thank you for sending us your work!

Sincerely,
John Wallingford
Monitoring Editor
Molecular Biology of the Cell

Dear Prof. weng,

The review of your manuscript, referenced above, is now complete. The Monitoring Editor has decided that your manuscript requires minor revisions before it can be published in Molecular Biology of the Cell, as described in the Monitoring Editor's decision letter above and the reviewer comments (if any) below.

A reminder: Please do not contact the Monitoring Editor directly regarding your manuscript. If you have any questions regarding the review process or the decision, please contact the MBoC Editorial Office (mboc@ascb.org).

When submitting your revision include a rebuttal letter that details, point-by-point, how the Monitoring Editor's and reviewers' comments have been addressed. (The file type for this letter must be "rebuttal letter"; do not include your response to the Monitoring Editor and reviewers in a "cover letter.") Please bear in mind that your rebuttal letter will be published with your paper if it is accepted, unless you have opted out of publishing the review history.

Authors are allowed 15 days to submit a revision. If this time period is inadequate, please contact us immediately at mboc@ascb.org.

In preparing your revised manuscript, please follow the instruction in the Information for Authors (www.molbiolcell.org/info-for-authors). In particular, to prepare for the possible acceptance of your revised manuscript, submit final, publication-quality figures with your revision as described.

To submit the rebuttal letter, revised version, and figures, please use this link (please enable cookies, or cut and paste URL):
Link Not Available

MBoC offers the option to publish your work with immediate open access. Open access can increase the discoverability and usability of your research. If you would like to publish your paper with immediate open access but did not select this option during initial submission, please contact us at mbc@ascb.org..

Authors of Articles and Brief Communications whose manuscripts have returned for minor revision ("revise only") are encouraged to create a short video abstract to accompany their article when it is published. These video abstracts, known as Science Sketches, are up to 2 minutes long and will be published on YouTube and then embedded in the article abstract.

Science Sketch Editors on the MBoC Editorial Board will provide guidance as you prepare your video. Information about how to prepare and submit a video abstract is available at www.molbiolcell.org/science-sketches. Please contact mboc@ascb.org if you are interested in creating a Science Sketch.

Thank you for submitting your manuscript to MBoC. Please do not hesitate to contact this office if you have any questions.

Sincerely,

Eric Baker
Journal Production Manager
MBoC Editorial Office
mbc@ascb.org

Reviewer #1 (Remarks to the Author):

Gu et al. have identified a novel Drosophila protein, Moat, as an important regulator of junctional Bazooka/Par3 levels during early Drosophila development. Apical constriction is a major cell shape change during tissue folding that involves the coordinated action of apical contractile myosin and adherens junctions. A gradient of myosin activity has been observed during ventral furrow formation, but the relationship between adherens junction levels and apical constriction remains unclear. Using high quality live imaging and detailed analysis, the authors show that Moat downregulates adherens junctions by inhibiting cortical clustering of Bazooka/Par3. The data are very clear and compelling. The study provides valuable insights into the role of tissue-scale distribution of adhesion levels in patterning apical constriction during tissue morphogenesis. Discussion of a potential mechanism for the function of Moat would enhance the impact of the paper.

Major comments
1. What kind of protein does Moat encode? This is the first report of the gene, but the authors did not provide information about the protein's structure. I searched the CG number of the gene on Flybase and found that the protein actually does not contain any functional domains. Still, since this is the first report, it will be informative to provide some more details about the protein, e.g., protein size, lack of specific domains, and whether it is conserved in other animals.

2. The study presents ample data to support the important role of Moat in regulating junctional Bazooka levels. However, the discussion is very short and the authors did not discuss enough about the potential mechanisms by which Moat regulates junctional Bazooka levels. A more detailed discussion of the possibilities would strengthen the study. The result paragraph for Fig.9 could actually be moved to Discussion.

Minor comments

1. It's not clear what kind of genetic screen was done to identify CG14427. Was it a screen using existing deficiencies, mutants, or RNAi? Provide a brief description in the text or in Methods.
2. Provide the sequence information for the Moat2A CRISPR mutant. A cartoon diagram with a deletion region along with the full sequence information would be helpful.
3. Give a brief description of the stomodeum in the text. Movie 2 has a label for the stomodeum, but no other figures show the structure, and it will be helpful for readers who are not familiar with it. Also, provide a citation (Movie 2) for "Stomodeum internalization is initiated later then ectoAMG."
4. Text for Fig. 4. "Loss of moat alone does not change lateral ectoderm and ventral furrow apical membrane morphology significantly." The experiment was for luciferase RNAi in the moat mutant background, not moat mutant alone. It would be more accurate to say that. It would also be clearer to add "compared to WT" to the sentence. Similarly, in the next sentence, "In moat mutants, lateral ectoderm still shows smooth apices with well-defined cell boundaries and ventral furrow cells still show small membrane blebs with invisible cell boundaries", it would be clearer to add "as in WT".
5. Figures should be displayed in the same order as they are described in the text. Currently, Fig.9 comes before Fig.8 in the text. Fig.9 seems to be more appropriate for Discussion.
6. All figures are missing scale bars. Add scale bars to SEM and confocal images.
7. Figures
- Fig. 5A and B right panels: Are these single sections or maximum projection as in the whole embryo images?
- In Fig. 5E and G: Provide time points in the panel.
- Fig. 9. It would be helpful to have a diagram of an embryo with VF and ectoAMG indicated.
- Fig. S1B. Provide a scale bar for the heat map.
8. Figure legends:
- Fig.1E: Do the solid lines show epithelial folds as in D? Indicate.

- Fig. 4D: Give the full name of Luci RNAi here or in the text.
- Fig.8. What is the marker of these images? Provide it in the legend. Also, provide the time unit (minute).
- Fig. S1B: The top image (early stage 5) is an en face view of the apical surface and the other three panels are z-sections along the apical/basal axis. Mention this in the legend.
- Fig. S2. The title says "giant gene expression pattern", but the figure shows immunostaining data, i.e., protein. Also, it would be helpful to indicate that the embryo in A is a ventrolateral view and B is a ventral view.

Reviewer #2 (Remarks to the Author):

Tissue folding is a key morphogenetic process during embryonic development, and it needs to be precisely controlled in time and space in order to pattern organs correctly. The Drosophila ventral furrow has been an important model system for understanding how cell fate patterning, cell behaviors (e.g. apical constriction and adhesion), and mechanics are coordinated to produce proper tissue folding. Here, Weng and colleagues contribute to the study of Drosophila ventral furrow formation by identifying a gene, moat, which when mutated causes ectopic furrow formation in the extreme anterior end of the embryo. The authors provide evidence that moat is somehow involved in the regulation of adherens junctions downstream of snail, though the mechanism is not explored in detail.
Overall, the manuscript is clearly written and easy to follow, and the results are interesting. There is much work left to do to understand what moat actually does, but that's reasonable considering that this is the first report of a newly discovered gene. I anticipate that most of my concerns below should be readily address through straightforward revisions.
• The manuscript largely consists of a detailed description of the moat mutant phenotype. A detailed investigation of moat's mechanism of action is clearly beyond the scope of this study, and I'm sure the authors will pursue that in future publications. But I'd have liked to see them at least speculate about what moat might actually do. What kind of protein does it encode? What domains does it have? Does it have homologs outside of flies? Do moat mutants have defects in other tissues whose folding involves AJ regulation, such as the dorsal folds? Answering these questions in the Discussion, even in a somewhat speculative way, would strengthen the paper.

• The simplest explanation for anteriorly extended apical constriction in the moat mutants would be a patterning defect. The authors try to rule this out by looking at Gt and Hkb, and indeed expression of those gap genes looks normal. But I think they need to also look directly at Snail expression (ideally both mRNA and protein), to confirm that moat mutants do not have ectopic furrowing due to ectopic Snail. The only image of Snail is in Figure 5A, and there it does indeed look (by eye) as though there might be excess Snail in the anterior. This needs to be examined carefully and quantified.

• Several figures need better quantification:
o In Figure 3, localization of Sqh::mCherry is shown, and based on these images, the authors claim that moat mutants have higher levels of "contractile myosin." To claim higher levels, the levels need to be measured and compared in a statistically robust way. To claim that any ectopic myosin is contractile, additional experiments would be needed, for example measuring apical tension using laser cutting. If the authors don't want to do those experiments, they could instead soften their claims about contractility.
o Quantification is also missing for the first half of Figure 5, for which the authors make strong claims about differences in intensity based only on single images.

• The authors also need to soften the claims that they make based on EM images presented in Figure 4. These images are necessarily static, and while I accept that an expert may be able to hypothesize whether myosin is active based on these images, to claim that morphology directly reports on myosin activity is an overinterpretation. The authors statement in the title of Figure 4, "Altered myosin activity contributes to the ectopic apical constriction in moat mutant ectoAMG", is far too strong. A more accurate legend would be "morphology of moat mutant ectoAMG is consistent with ectopic apical constriction." To claim that altered myosin activity contributes to altered apical constriction, perturbations of myosin activity (for example using ROCK inhibitors) would be required.

• Does Fig. 9 depict experimental data, or is it illustrating a hypothesis? I suspect the latter, and if so, I would strongly discourage the authors from depicting hypotheses in the form of graphs that could be mistaken for real data. A cartoon illustration of the model would be more appropriate here, or alternatively, a "model figure" could be omitted since the data are fairly descriptive and don't imply a specific model for how moat is acting.

Reviewer #1 (Remarks to the Author):

Gu et al. have identified a novel Drosophila protein, Moat, as an important regulator of junctional Bazooka/Par3 levels during early Drosophila development. Apical constriction is a major cell shape change during tissue folding that involves the coordinated action of apical contractile myosin and adherens junctions. A gradient of myosin activity has been observed during ventral furrow formation, but the relationship between adherens junction levels and apical constriction remains unclear. Using high quality live imaging and detailed analysis, the authors show that Moat downregulates adherens junctions by inhibiting cortical clustering of Bazooka/Par3. The data are very clear and compelling. The study provides valuable insights into the role of tissue-scale distribution of adhesion levels in patterning apical constriction during tissue morphogenesis. Discussion of a potential mechanism for the function of Moat would enhance the impact of the paper.

Major comments

1. What kind of protein does Moat encode? This is the first report of the gene, but the authors did not provide information about the protein's structure. I searched the CG number of the gene on Flybase and found that the protein actually does not contain any functional domains. Still, since this is the first report, it will be informative to provide some more details about the protein, e.g., protein size, lack of specific domains, and whether it is conserved in other animals.

We thank the reviewer for raising this point. It makes sense to include the basic information even if there aren't any recognizable domains. We have revised the text to add the following information to the first section of the manuscript and included an AlphaFold predicted structure in the supplemental figure:
"Moat protein is expressed in a similar pattern as its mRNA (Fig. S1C). Moat protein consists of 252 amino acids and its homologs are found in the Brachyceran suborder of Dipteran insects. Although there isn't any recognizable functional domain, Moat protein appears to associate with cell membrane or cortex (Fig. S1C-D)."

2. The study presents ample data to support the important role of Moat in regulating junctional Bazooka levels. However, the discussion is very short and the authors did not discuss enough about the potential mechanisms by which Moat regulates junctional Bazooka levels. A more detailed discussion of the possibilities would strengthen the study. The result paragraph for Fig.9 could actually be moved to Discussion.

We thank the reviewer for the suggestions to improve the manuscript. We added a paragraph to the Discussion section as shown below. We highlighted our observation that Baz puncta at the junctional cortex are bigger and more intense in the *moat* mutants and discussed the possible mechanisms by which Moat could regulate cortical Baz puncta formation.

We are confused by the comment "result paragraph for Fig. 9 could be moved to Discussion." Fig. 9 has always been in the Discussion section of our manuscript. However, we did revise the figure to make it more intuitive.

" Moat appears to facilitate Sna in downregulating junctional Baz by reducing the junctional localization of Baz. The junctional puncta of Baz appear to be bigger and more intense in the *moat* mutants, suggesting Moat may limit the size and density of Baz puncta at junctional cortex. The condensation of Baz proteins into punctate structures at the cortex has been shown to be driven by liquid-liquid phase separation (Liu et al., 2020). Many mechanisms collectively contribute to such puncta formation, including the oligomerization and membrane association of Baz proteins, interaction of Baz with other cortical polarity proteins, and microtubule-dependent transport of Baz puncta (Harris, 2017). Our data using endogenously tagged Moat show that Moat associates with the cell membrane or cortex (Fig. S1C). This raises the possibility that Moat may interact with cortically localized Baz or its regulators, thereby reducing the interactions required for puncta formation at the junctional cortex. Alternatively, Moat may change the local membrane composition and make the junctional cortex less favorable for Baz to form puncta."

Minor comments

1. It's not clear what kind of genetic screen was done to identify CG14427. Was it a screen using existing deficiencies, mutants, or RNAi? Provide a brief description in the text or in Methods.

We agree that it's helpful to include the type of genetic screen in the text and have revised accordingly: "To understand tissue boundary formation, we identified an uncharacterized gene *CG14427* through a **compound-chromosome** genetic screen looking for mutants with disrupted organization of ventral furrow."

2. Provide the sequence information for the Moat2A CRISPR mutant. A cartoon diagram with a deletion region along with the full sequence information would be helpful.

A diagram has been added to Figure S1 to illustrate the allele.

3. Give a brief description of the stomodeum in the text. Movie 2 has a label for the stomodeum, but no other figures show the structure, and it will be helpful for readers who are not familiar with it. Also, provide a citation (Movie 2) for "Stomodeum internalization is initiated later than ectoAMG."

We appreciate the reviewer's suggestion regarding the need for clarity. There is no existing citation for the stomodeum being a separate and later invagination because we are the first to observe this. We have revised the text below to clarify this point. Since the majority of the stomodeum cells are not visible on the ventral side of the

embryo, we have added a midsagittal view of a blastoderm embryo in Fig. 1A to show the position of the stomodeum as defined by our study."

“**This is in contrast to the past description that ectoAMG participates in the triangle-shaped stomodeum invagination** (Hartenstein et al., 1985; Reuter and Leptin, 1994a; Technau and Campos-Ortega, 1985). **Our live imaging data show that the triangular stomodeum invagination is a separate event: it consists of cells anterior to ectoAMG and is initiated later than the circular ectoAMG invagination (Movie 2). The triangle-shaped invagination also features much higher levels of adherens junctions compared to ectoAMG, making the two tissues easily distinguishable.** Altogether, our data indicate that the internalization of wild type ectoAMG is a morphogenetic event different from that of ventral furrow and stomodeum in both morphology and timing.”

4. Text for Fig. 4. "Loss of moat alone does not change lateral ectoderm and ventral furrow apical membrane morphology significantly." The experiment was for luciferase RNAi in the moat mutant background, not moat mutant alone. It would be more accurate to say that. It would also be clearer to add "compared to WT" to the sentence. Similarly, in the next sentence, "In moat mutants, lateral ectoderm still shows smooth apices with well-defined cell boundaries and ventral furrow cells still show small membrane blebs with invisible cell boundaries", it would be clearer to add "as in WT".

We thank the reviewer for the suggestion and revised the manuscript accordingly: “In ***moat* embryos with control luciferase RNAi (*Luci* RNAi), lateral ectoderm and ventral furrow do not show significant changes in apical membrane morphology compared to wild type:** lateral ectoderm still shows smooth apices with well-defined cell boundaries and ventral furrow cells still show small membrane blebs with invisible cell boundaries”

5. Figures should be displayed in the same order as they are described in the text. Currently, Fig.9 comes before Fig.8 in the text. Fig.9 seems to be more appropriate for Discussion.

Again, we are unsure about the exact meaning of this comment. Figure 9 does not precede Figure 8 in our manuscript, and it is already included in the discussion section.

6. All figures are missing scale bars. Add scale bars to SEM and confocal images.

All scale bars have now been added.

7. Figures

We appreciate the reviewer's comments and have revised accordingly.

- Fig. 5A and B right panels: Are these single sections or maximum projection as in the whole embryo images?

We revised the legend to clarify these.

- In Fig. 5E and G: Provide time points in the panel.

We added the time points.

- Fig. 9. It would be helpful to have a diagram of an embryo with VF and ectoAMG indicated.

We have revised Fig. 9 substantially to make things more clear.

- Fig. S1B. Provide a scale bar for the heat map.

We added the heat map scale bar to the figure.

8. Figure legends:
- Fig.1E: Do the solid lines show epithelial folds as in D? Indicate.

We added the information in the figure legend.

- Fig. 4D: Give the full name of Luci RNAi here or in the text.

We have changed the text to "embryos with control luciferase RNAi (*Luci* RNAi)".

- Fig.8. What is the marker of these images? Provide it in the legend. Also, provide the time unit (minute).

We added "E-Cad::GFP" labels to the figure and changed the time labeling to indicate the unit.

- Fig. S1B: The top image (early stage 5) is an en face view of the apical surface and the other three panels are z-sections along the apical/basal axis. Mention this in the legend.

We added such information to the legend.

- Fig. S2. The title says "giant gene expression pattern", but the figure shows immunostaining data, i.e., protein. Also, it would be helpful to indicate that the embryo in A is a ventrolateral view and B is a ventral view.

We changed the “gene expression” to “protein expression” and added information about the views.

Reviewer #2 (Remarks to the Author):

Tissue folding is a key morphogenetic process during embryonic development, and it needs to be precisely controlled in time and space in order to pattern organs correctly. The Drosophila ventral furrow has been an important model system for understanding how cell fate patterning, cell behaviors (e.g. apical constriction and adhesion), and mechanics are coordinated to produce proper tissue folding. Here, Weng and colleagues contribute to the study of Drosophila ventral furrow formation by identifying a gene, moat, which when mutated causes ectopic furrow formation in the extreme anterior end of the embryo. The authors provide evidence that moat is somehow involved in the regulation of adherens junctions downstream of snail, though the mechanism is not explored in detail.
Overall, the manuscript is clearly written and easy to follow, and the results are interesting. There is much work left to do to understand what moat actually does, but that's reasonable considering that this is the first report of a newly discovered gene. I anticipate that most of my concerns below should be readily address through straightforward revisions.

• *The manuscript largely consists of a detailed description of the moat mutant phenotype. A detailed investigation of moat's mechanism of action is clearly beyond the scope of this study, and I'm sure the authors will pursue that in future publications. But I'd have liked to see them at least speculate about what moat might actually do. What kind of protein does it encode? What domains does it have? Does it have homologs outside of flies? Do moat mutants have defects in other tissues whose folding involves AJ regulation, such as the dorsal folds? Answering these questions in the Discussion, even in a somewhat speculative way, would strengthen the paper.*

We thank the reviewer for the comments. This has been addressed in response to reviewer 1’s comments. We added information about Moat protein and added more discussion about possible mechanism of how Moat may regulate junctional Baz.

• *The simplest explanation for anteriorly extended apical constriction in the moat mutants would be a patterning defect. The authors try to rule this out by looking at Gt and Hkb, and indeed expression of those gap genes looks normal. But I think they need to also look directly at Snail expression (ideally both mRNA and protein), to confirm that moat mutants do not have ectopic furrowing due to ectopic Snail. The only image of Snail is in Figure 5A, and there it does indeed look (by eye) as though there might be excess Snail in the anterior. This needs to be examined carefully and quantified.*

We appreciate the Snail hypothesis raised by the reviewer. However, we do not believe that ectopic Snail expression would explain the *moat* mutant phenotype. An ectopic increase in Snail expression would result in even less junctional Baz compared to wild type, which is contrary to the elevated junctional Baz phenotype observed in *moat* mutants.

Furthermore, we have examined a large number of wild type and *moat* mutant embryos immunostained for Snail, and we have not observed any consistent differences in Snail levels between the mutants and wild type embryos. Although there may be variation among individual embryos, which is common with immunostaining, overall, mutant and wild type embryos appear comparable.

Lastly, we observed that *moat* mutants are viable. Although it has not been tested thoroughly that ectoAMG is essential for viability, this result would suggest ectoAMG most likely is specified normally in *moat* mutants.

However, we agree that it would be helpful to present Snail protein expression in mutants before Fig. 5. We added images showing Snail staining in mutant embryos in Fig. 1.

• Several figures need better quantification:
1) In Figure 3, localization of Sqh::mCherry is shown, and based on these images, the authors claim that moat mutants have higher levels of "contractile myosin."
To claim higher levels, the levels need to be measured and compared in a statistically robust way. To claim that any ectopic myosin is contractile, additional experiments would be needed, for example measuring apical tension using laser cutting. If the authors don't want to do those experiments, they could instead soften their claims about contractility.

We thank the reviewer for raising the concern about myosin contractility. Our primary intention is not to assert contractility, and in fact we intended to show myosin activities in *moat* mutant ectoAMG is insufficient to account for the ectopic folding behavior without the elevated levels of junctional Baz. We examined the myosin activities in ectoAMG in order to give a thorough description of *moat* mutant phenotype. While we considered various terminologies, we chose the term "contractile myosin" to describe the intense and pulsatile cortical myosin population to maintain consistency with the previous studies cited in the manuscript (Heer et al., 2017; Vasquez et al., 2016; Xie and Martin, 2015). These studies have established that the intensity of these bright and pulsatile myosin signals in the fly ventral furrow is indicative of myosin contractility. **We have revised the manuscript to clarify that "contractile myosin" specifically refers to this population of intense, pulsatile, cortical myosin.**

Since contractility is not our primary focus, we did not perform laser cutting experiments. Instead, we presented data supporting that the ectopic ectoAMG myosin appears to be contractile similar to the myosin in ventral furrow. For example, our live imaging data show that the myosin in *moat* mutant ectoAMG are intense, pulsatile and cortical, characteristics that are consistent with contractile myosin as established in previous studies.

Importantly, we showed the ectopic folding of mutant ectoAMG requires cta and T48 pathways which have been well characterized to activate contractile myosin in normal ventral furrow through recruiting RhoGEF2. Although it is not impossible, it seems unlikely that cta and T48 have an unknown function that accumulates intense myosin without contractility in the mutants. **We revised the manuscript to reflect this point and also replaced "contractile myosin" with other descriptions such as "myosin activities" for *moat* mutant ectoAMG.**

As for measurement, we don't believe it would be accurate or informative to measure and compare myosin in wild type and *moat* mutant ectoAMG because apical constriction does not occur in the former but is robust and leads to tissue folding in the latter. Myosin will be more concentrated in constricted cells and the measurement will surely be higher in the *moat* mutant embryos. We will get the results supporting that myosin is slightly higher in mutant ectoAMG compared to wild type, but it won't be informative and could be misleading. This is one of the reasons we did the scanning EM in figure 4 to evaluate myosin activities without apical constriction.

2) Quantification is also missing for the first half of Figure 5, for which the authors make strong claims about differences in intensity based only on single images.

Regarding Figure 5, the first half aims to illustrate the appearance of the punctate form of junctional Baz and adherens junctions, as well as the apparent consequences of downregulation in the mesoderm. Two reasons account for the apparent lack of quantification, which we have addressed in the revised manuscript:

1) The differences between mesoderm and ectoderm in wild-type embryos have already been quantified in one of our previous studies, referenced in the manuscript. We revised the manuscript to make the citation more clear.

2) The images in the first half of Figure 5 are derived from fixed embryos to demonstrate both mesoderm and ectoderm. However, quantifying these phenomena using fixed embryos is often unreliable due to individual differences between immunostained embryos and the challenge of obtaining embryos of precisely the same timing, as downregulation occurs over a short span of 10-15 minutes. Hence, we conducted quantification of the same processes using live imaging data to compare wild-type and mutant embryos, presented in the second half of the figure. We have made this distinction clearer in the revised manuscript. We revised the manuscript to make this more clear.

• The authors also need to soften the claims that they make based on EM images presented in Figure 4. These images are necessarily static, and while I accept that an expert may be able to hypothesize whether myosin is active based on these images, to claim that morphology directly reports on myosin activity is an overinterpretation. The authors statement in the title of Figure 4, "Altered myosin activity contributes to the ectopic apical constriction in moat mutant ectoAMG", is far too strong. A more accurate legend would be "morphology of moat mutant ectoAMG is consistent with ectopic apical constriction." To claim that altered myosin activity contributes to altered apical

constriction, perturbations of myosin activity (for example using ROCK inhibitors) would be required.

We appreciate the reviewer's comments. We wish to clarify that we did not intend to assert that apical morphology directly reflects myosin activity. The correlation between apical morphological changes and myosin activity has been well established in previous studies, which we have cited. The aim of the figure is to offer an alternative comparison of myosin activities between wild-type ectoAMG, mutant ectoAMG, and the ventral furrow. As mentioned earlier, live imaging data come with their own limitations in evaluating myosin activities, so we sought to present the comparison using an independent approach. However, we acknowledge that the figure title was an oversight on our part and does not accurately convey our conclusion. We thank the reviewer for suggesting a revised figure title, and we have accordingly changed it to "*moat* mutant ectoAMG does not resemble ventral furrow in the complete cell rupture phenotype upon loss of junctions."

• Does Fig. 9 depict experimental data, or is it illustrating a hypothesis? I suspect the latter, and if so, I would strongly discourage the authors from depicting hypotheses in the form of graphs that could be mistaken for real data. A cartoon illustration of the model would be more appropriate here, or alternatively, a "model figure" could be omitted since the data are fairly descriptive and don't imply a specific model for how moat is acting.

We appreciate the reviewer's suggestion to change the graph to a diagram. This is a good idea, as a diagram is often more intuitive and helpful. Therefore, we have created a diagram to replace the graph. Additionally, we revised the figure title since the old title may be another reason for the reviewer’s confusion.

RE: Manuscript #E24-04-0177R
TITLE: "A novel protein Moat prevents ectopic epithelial folding by limiting Bazooka/Par3-dependent adherens junctions"

Dear Dr. Weng,

I'm pleased now to accept this paper for Publication in MBoC!

Sincerely,
John Wallingford
Monitoring Editor
Molecular Biology of the Cell

Dear Prof. Weng:

Congratulations on the acceptance of your manuscript! Thank you for choosing to publish your work in Molecular Biology of the Cell (MBoC).

Within 10 days, an unedited PDF of your manuscript will be published on MBoC in Press, an early release version of the journal. The date your manuscript appears on this site is the official publication date.

Your copyedited and typeset manuscript will be scheduled for publication in the next available issue of MBoC. Our production team will notify you when the page proofs of your paper are ready for your review.

MBoC offers the option to publish your paper with immediate open access. Open access can increase the discoverability and usability of your research. If you would like to publish your paper with immediate open access but did not select this option during initial submission, please contact the MBoC Editorial Office (mbc@ascb.org).

We invite you to submit images related to your accepted manuscript to be considered for the journal cover.
Cover images must contain color, TIF Format
Size image at 7.4" wide x 5.85" deep
RGB and CMYK color (please submit two separate files, one in each format)
300 dpi resolution

Please also send a 1-paragraph description of the image that a general audience could understand. Include with the caption the name and institution of the individual(s) the image should be attributed to. Please provide this description in a Word file. You can send the file via email or by your favorite file transfer service to mboc@ascb.org.

Authors of Articles and Brief Communications are also encouraged to create a short video abstract to accompany their article when it is published. These video abstracts, known as Science Sketches, are up to 2 minutes long and will be published on YouTube and then embedded in the article abstract. Science Sketch Editors on the MBoC Editorial Board will guide you as you prepare your video. Information about how to prepare and submit a video abstract is available at www.molbiolcell.org/science-sketches. Please contact mboc@ascb.org if you are interested in creating a Science Sketch.

We look forward to publishing your paper in MBoC.
Sign up for MBoC's new content alerts here https://tinyurl.com/3bcsyk3e

Sincerely,

Eric Baker
Journal Production Manager
MBoC Editorial Office
mbc@ascb.org
